# Trends over the past 15 years in long-term care in Switzerland: a comparison with Germany, Italy, Norway, and the United Kingdom

**DOI:** 10.1186/s12877-024-05195-8

**Published:** 2024-07-23

**Authors:** Clara Lussi, Jerome Bickenbach, Rune Halvorsen, Carla Sabariego

**Affiliations:** 1 Swiss Paraplegic Research, Guido A. Zäch-Strasse 4, Nottwil, 6207 Switzerland; 2 Faculty of Health Sciences and Medicine, University of Lucerne, Frohburgstrasse 3, Lucerne, 6002 Switzerland; 3 Oslo Metropolitan University, Stensberggata 26, Oslo, 0170 Norway; 4 Center for Rehabilitation in Global Health Systems, World Health Organization Collaborating Center, University of Lucerne, Frohburgstrasse 3, Lucerne, 6002 Switzerland

**Keywords:** Long-term care, Public policy, Comparative study, Switzerland, Nursing services

## Abstract

**Background:**

The demographic changes affecting Switzerland and other European countries, including population ageing, will continue to challenge policymakers in building accessible, affordable, comprehensive and high-quality long-term care (LTC) systems. The purpose of this paper is to investigate how Switzerland’s LTC system compares to other European countries, in order to inform how to respond to the increasing need for LTC. We carried out a descriptive study using secondary data from key national and international organizations.

**Methods:**

By comparing the financing, workforce, service delivery and need for LTC in Switzerland, Germany, Italy, Norway and the United Kingdom, we described similarities and differences in these five European countries between 2005-2019. Thirty-three indicators within five domains were analysed: (1) Population statistics and health expenditure, (2) Need for LTC, (3) LTC financing, (4) LTC service delivery, and (5) LTC workforce.

**Results:**

Switzerland has the highest life expectancy in comparison to the other four high-income countries. However, similarly to other countries, the years lived with disability are increasing in Switzerland. Switzerland’s public expenditure on LTC as a share of GDP is lower than that of Norway and Germany, yet out-of-pocket expenditure on LTC is highest in Switzerland. Switzerland has the highest proportion of persons receiving formal LTC both in institutions and at home. Switzerland has had the most pronounced increase in the proportion of over 65-year-olds receiving LTC at home. Even though more than fourfold more persons receive care at home, Switzerland still has more workforce in LTC institutions than in home-care. In comparison to Germany and the UK, Switzerland has a lower number of informal carers as a proportion of 50-year-olds and over, as well as fewer nationally available services for informal carers compared to Germany, Italy, Norway and the UK.

**Conclusions:**

Our comparative study corroborates the importance of improving the affordability of LTC, continuing to support the movement towards home care services, improving the support given to both the professional workforce and informal carers, and improving the amount and quality of LTC data. It also provides a valuable contrast to other European countries to support evidence-informed policymaking.

**Supplementary Information:**

The online version contains supplementary material available at 10.1186/s12877-024-05195-8.

## Introduction

As with other high-income countries (HICs), Switzerland is experiencing population ageing [1]. The population of over 80-year-olds is predicted to grow 2.4 times, from 0.46 million in 2020 to 1.11 million by 2050 [2]. The second most rapidly growing population category is the over 65-year-olds, predicted to grow 1.63 times by 2050 [2]. Not only are people living longer, but they are also living with higher levels of disability [3]. This is due to the rising prevalence of non-communicable diseases and the growing gap between life expectancy and healthy life expectancy, also known as ‘years lived with disability’ [3].

The demographic changes affecting Switzerland and other European countries will challenge policy-makers. These countries’ health and welfare systems will need to ensure sufficient high-quality long-term care (LTC) for the growing number of persons with LTC needs while also providing protection against the resulting financial burden [4, 5]. The COVID-19 pandemic also highlighted gaps and vulnerabilities in the LTC system and the fragmentation between the health system, LTC system and social welfare system [6, 7]. Providing person-centered, integrated care and LTC are also crucial action areas within the World Health Organization’s (WHO) Global Strategy and Action Plan on Ageing and Health and the United Nation’s (UN) Decade of Healthy Ageing [8, 9].

The WHO defines LTC as *“a broad range of personal*,* social and medical services and support that ensure people with*,* or at risk of*,* a significant loss of intrinsic capacity (due to mental or physical illness and disability)*,* can maintain a level of functional ability consistent with their basic rights and human dignity”* [10]. The WHO delineates a separate LTC system which lies between healthcare systems and social welfare systems and encompasses assistive personal care, social participation support, as well as caregiver support [10]. In reality, LTC systems often span between healthcare systems and social welfare systems [10]. While social welfare systems provide services such as cash benefits for both care users and caregivers, health care services include disease management and care coordination [10]. These services may be provided formally in community and care facilities as well as informally through persons within the close social circle of the care user (family, friends, neighbors) [10]. In this manuscript we use the term LTC to encompass a range of services, including institutional, home and informal care. Although, the LTC services formally available to residents of a country are different, depending on local health and welfare policies.

The challenges of LTC are similar between European countries. LTC is expensive and public spending on healthcare and social welfare systems is projected to continuously increase [4, 5, 11]. Similarly, LTC is expensive for individuals, which is often a barrier to access to the care needed [11]. Many countries, including Switzerland, also face a shortage in the care workforce [11]. Informal care is often used as a form of easily accessible care, but this also has consequences, as it often takes caregivers out of work and can have a negative impact on their health and retirement benefits [11, 12]. Finally, quality of care, including avoiding fragmentation of care, is often not comparable within countries and risks being neglected as demand increases [11]. For Switzerland to face the demographic shift successfully, LTC must become financially sustainable, accessible, equitable, of high quality, and there must be enough care workers to provide care.

The current Swiss response to the challenges of LTC is delineated in the national health strategy, Health2030 [13]. The Health2030 strategy is the health policy agenda of the Swiss Federal Council from the years 2020 to 2030. One of the challenges delineated in this agenda is “Demographic and Social Trends” [13]. The two objectives within this challenge are [1] safeguarding care and funding as well as [2] ensuring healthy ageing [13]. Additionally, key areas of qualitative and quantitative impact for the ageing population are ensuring enough health workforce, ensuring financing to support, and improving the quality of the services [13]. Switzerland has clear goals to overcome the challenges posed by the demographic shift, and these must be continuously tracked and assessed.

Switzerland frequently relies on cross-country comparative studies to inform policymaking. For instance, the Swiss Health Observatory has recently published an international comparison of the experiences of the Swiss population aged 65 years and older with the health care system [14]. The Swiss Federal Office of Statistics has also recently published indicators on health in European comparison [15]. Cross-country comparison may provide valuable information to decision-makers, by learning from structural and resource differences that may be linked to differences in the provision and performance of health care [16, 17]. International organizations such as the OECD, WHO, European Observatory on Health Systems and Policies, have published a plethora of reports that include international comparisons of long-term care systems. However, they often are limited to either one aspect such as financing or workforce. Additionally, there have not been any reports published with specific policy recommendations for Switzerland. As each system is so different and stands in different points of development, it was of interest to take a broad approach and give recommendations to Switzerland specifically. In 2016, the Swiss Federal Council published a detailed report, describing the long-term care system and its challenges [18]. The findings from the report aimed to open questions for public debate on future goals and measures to be taken to sustainably ensure adequate provision and financing of long-term care. It highlighted a few insights from some of these international reports, however, did not take an analytical approach, suggesting concrete directions. This manuscript, based on existing frameworks, a selected list of indicators and comparator countries, adds to the literature by providing an updated overview and informing on the specific gaps and policy considerations in the Swiss long-term care system.

The objective of this paper is, by comparing LTC in Switzerland to Germany, Italy, Norway, and the United Kingdom, to inform policy-making so as to respond to the challenge of the increasing need for LTC in Switzerland. The scope of this analysis is limited to the elderly population due to the extreme heterogeneity and incompleteness of disability data, although it is clear that LTC is a service that should target persons with care needs of all ages. Specifically, we aim to identify trends over the past 15 years in the [1] need for LTC, [2] LTC financing, [3] LTC service delivery, and [4] LTC workforce. The trends will be understood in light of current policies and the similarities and differences in the country’s response to the changing needs. The lessons learned for Switzerland can apply to other HICs facing comparable challenges.

## Materials and methods

### Selection of study and comparator countries

We examined Switzerland and four comparator HICs, Germany, Italy, Norway and the UK. The comparator countries were chosen because they are all high-income European countries with similar demographic trends in ageing populations, yet with differently structured healthcare, welfare and LTC systems [19, 20]. The highlights of each country’s care regimes and welfare systems that make them interesting for this comparison are as follows: [1] Germany is one of the rare countries that has a separate LTC insurance, [2] Italy is the most aged country in Europe, meaning it is already facing many of the challenges that may confront other countries in coming years. It also has a cash benefit available to persons with LTC needs without means-testing, [3] Norway has a tax-based LTC funding system which ensures over-proportional public funding and low out-of-pocket spending, [4] the UK has national means-testing and eligibility criteria for accessing LTC as well as relatively low spending and high performance.

In order to give more background on the long-term care systems of each country and to contextualize them, two key issues are highlighted in Table 1: the eligibility criteria for LTC and the services publicly available in each country. In Switzerland, anyone is eligible to access LTC services, but reimbursement through health insurance is only available if the services have been prescribed by a physician. The reoccurring access variability among Swiss regions also exists in LTC due to the responsibility residing within cantons. Germany is an example of a country where the eligibility criteria for LTC are nationally defined: to be eligible for LTC, the applicant must be in need of care for at least six months and go through a nationally standardized evaluation system. Regional differences exist in Switzerland, Italy, Norway and the UK. In all countries, health professionals or health units decide the eligibility of a person for LTC.

All countries have similar forms of institutional care and professional home care, although they may vary in forms and titles (Table 1). Of note is Norway’s support person, which is a service that has an additional eligibility criterion of having unmet social needs. Interestingly, persons over the age of 67 are rarely allowed a support person. In Switzerland an assistance contribution is available to persons who receive a ‘helplessness allowance’ (official translation of the Swiss social insurance *Hilflosenentschädigung*). Beneficiaries must need assistance in activities of daily living and live at home. This allows someone with high care needs to perform activities of daily living while remaining at home. What varies the most from country to country is the services available for informal home care. Germany and the UK have the most extensive services available for informal caregivers, including training and employment services. Switzerland is the only country without direct financial support for informal home carers, nationally.

**Table 1:** Eligibility criteria and long-term care services

Switzerland	Germany	Italy	Norway	United Kingdom
**Eligibility criteria for long-term care**				
Long-term care services are accessible to anyone, however in order for the medical services to be reimbursed, they must be prescribed by a doctor. The level of contribution of the health insurance is assessed by the long-term care providers using, most commonly, one of three instruments: RAI-RUG (Resident Assessment Instrument – Resource Utilization Group), the BESA (Bedarfsklärungs- und Abrechnungs-System) or the PLAISIR (Planification Informatisée des Soins Infirmiers Requis).	Services must be accessed through application to the statutory LTC-Insurance. Eligibility for insurance benefits is determined by a Medical Review Board, who must evaluate the applicant as in need of care for at least six months. The following categories are used to evaluate score the need of care: mobility (10%), cognitive and communicative abilities (15%), behaviour patterns and psychological problems (15%), level of self-sufficiency (40%), health restrictions, demands and stress of treatment (20%) and structure of everyday life and social contacts (20%). Patients are categorized into one of five care levels. The amount of benefits received depends on the care level.	Italy does not have a single national legal definition to refer to for persons in need of care (disabled, handicapped and non-autonomous). The assessment for need of care is also the responsibility of local health units. For these reasons, eligibility for need of care is highly variable. All processes start with an application to the local health unit, which is followed by a needs assessment by either a medical commission or a general practitioner.	Eligibility criteria are set by the municipalities. The needs assessment varies by municipalities, but are all based on the IPLOS pseudonymous statistics registration for health and care, which obliges municipalities to register extensive information on the functional capabilities of each patient. The decision is taken by the provider or the municipality’s independent assessment unit and is supported by the patient’s GP’s recommendations with regard to the appropriate level of care.	National eligibility criteria were introduced by The Care Act in 2014, however there is still great variation across the country. Local authorities must assess anyone who appears to require care and support, however eligibility is often dependent on what funding is available. The assessment should be performed by a council or external agency. An individual will be eligible for care if their disability or illness prevents them from achieving two or more daily activities that significantly impact their well-being.
**Publicly available long-term care services**				
1. Institutional care a. Nursing homes with a high number of nursing beds b. Old-age home with a low number of nursing beds 2. Professional home care a. Spitex home care b. Day and night clinics c. Assistance contribution 3. Informal home care a. Continued payment of wages in the event of short absences from work for carers b. Old age and survivors’ insurance credits for carers c. Some cantons have cash benefits for informal carers	1. Institutional care 2. Professional home care a. Home care b. Day and night clinics 3. Informal home care a. Cash-benefits to cover informal home care b. Free training courses for informal carers c. Short-term replacement care for when usual carers take holidays d. Statutory accident insurance and statutory retirement insurance for informal carers	1. Institutional care a. Residential institutions b. Semi-residential institutions 2. Professional home care 3. Informal home care a. Cash benefit titled companion allowance for persons with disabilities, regardless of age and economic status b. Paid care leave for carer of person with severe disability	1. Institutional care a. Nursing homes for high degree of medical care b. Sheltered houses for same services as nursing homes but usually for residents with fewer care needs 2. Professional home care a. Home care b. Support person to help fulfil the social needs of the patient* 3. Informal home care a. Financial support for care provided titled attendance benefit b. Respite support to prevent burn-out among carers, allow them to go on holidays and have a normal social life	1. Institutional care 2. Professional home care 3. Informal care a. Financial support for carers, titled The Carers Allowance b. Day-care services and short-term institutional respite care to give the carer breaks from caring c. Working-age employment support service so that carers can update their skills and knowledge level if they want to obtain employment while caring.

*Only available for under 67-year olds

### Selection of indicators

In order to understand the countries’ similarities and differences, as well as the 15-year trend in need of LTC, we selected five domains of indicators. The domains were selected based on the overlapping domains covered by the health system building blocks of the WHO framework for health systems [21] and the “Demographic and Social Trends” challenge within the Health2030 strategy of the Swiss Federal Council [13]. In order to contextualize country responses, general national statistics and estimates of the need for LTC are presented as additional domains of indicators as well. Thus, we selected 33 indicators within five domains: [1] Population statistics and health expenditure, [2] Need for LTC, [3] LTC financing, [4] LTC service delivery, and [5] LTC workforce. Table 2. presents the study aims and the associated indicators.

The first domain “Population statistics and health expenditure” was used to provide baseline information on the size and makeup of the countries’ populations, gross domestic product (GDP) and healthcare expenditure. The second domain then compared past and future trends in the need for LTC. We examined the past trends using data on life expectancy and healthy life expectancy at birth and the proportion of over 65-year-olds and under 64-year-olds receiving LTC in institutions and care at home. The future need was portrayed using population projections, the number of people to receive care in an institution, and the number of people to receive care at home. These indicators for the need for LTC support the comparison of all other indicators by presenting the actual demand for LTC. They also support the contextualization of the countries’ responses to the upcoming demographic shift by presenting the future demand.

Our subsequent domain of analysis was financing for LTC, examining total expenditure, public expenditure, and household out-of-pocket payments on LTC. Each of the LTC financing indicators was examined as a share of GDP, as a share of health expenditure and per capita. Per capita is always presented in the form of current prices with current purchasing power parity (PPP) in US dollars, sourced from OECD Statistics and last updated on December 6th, 2022 [22]. Unfortunately, the available data for the LTC financing indicators only include the health portion of expenditure. This excludes a large part of LTC expenditure, notably from the social welfare state. In order to extend the examination of trends into the future, we also included the projected public expenditure as a share of GDP. These indicators allow an examination of the similarities and differences in the extent of public intervention in the financing of LTC.

Next, we examined LTC service delivery, including the number of recipients in institutions and at home and the number of beds in institutions. These indicators present data on the most common forms of LTC in Europe and allow the investigation of the similarities and differences in the availability and use of services in the compared countries.

Finally, we analyzed the workforce. The formal sector was examined through data on the total number of nurses and personal carers, total nurses and personal carers working in LTC institutions, and total nurses and personal carers working in long-term home care. The informal sector was examined using data on the share of informal carers among the population aged 50 and over. For Norway, the matching data was only available for the share of informal carers among the population aged 16 and over. The workforce indicators enable the examination of the similarities and differences in the availability and mix of the countries’ formal and informal carers.

**Table 2:** Study aims and associated indicator selection

Aim	Indicator
**Trends over the past 15 years in need for LTC**	Life expectancy at birth
	Healthy life expectancy (HALE) at birth
	Proportion of 65-year-olds and over receiving long-term care in institutions
	Proportion of 65-year-olds and over receiving long-term care at home
	Proportion of 64-year-olds and under receiving long-term care in institutions
	Proportion of 64-year-olds and under receiving long-term care at home
	Population projections, in millions
	Number of people to receive care in an institution
	Number of people to receive care at home
**Trends over the past 15 years in LTC financing**	Total expenditure on (health-related) long-term care - *As share of GDP* - *As share of health expenditure* - *Per capita (current prices*,* current ppp*,* USD)*
	Public expenditure on (health-related) long-term care (government and compulsory schemes) - *As share of GDP* - *As share of health expenditure* - *Per capita (current prices*,* current ppp*,* USD)*
	Household out-of-pocket payments on (health-related) long-term care - *As share of GDP* - *As share of health expenditure* - *Per capita (current prices*,* current ppp*,* USD*
	Projected public expenditure on (health-related) long-term care, as % of GDP
**Trends over the past 15 years in LTC service delivery**	Long-term care recipients in institutions (other than hospitals), n (% of total population)
	Beds in institutional LTC facilities, n (per 1000 population aged 65 and over)
	Long-term care recipients at home, n (% of total population)
**Trends over the past 15 years in LTC workforce**	Total nurses and personal carers, n (% of population aged 65 and over)
	Total nurses and personal carers working in long-term care institutions, n (% of population aged 65 and over)
	Total nurses and personal carers working in long-term home care, n (% of population aged 65 and over)
	Proportion of informal carers among population aged 50 and over

*Abbreviations* GDP (Gross Domestic Product); LTC (Long-Term Care); PPP (Purchasing power parity); USD (United States Dollar)

### Data sources

Data for population statistics came from OECD Statistics [22] and the World Bank [23]. The data for country expenditure, insurance coverage, financing, service delivery, workforce, and need for LTC is mostly from the OECD [22, 24–27]. When data was not available for a certain country, additional data sources, both country-specific and international, were used to fill the gaps. Of course, the use of varying data sources reduces the comparability of the data. Nevertheless, the additional data sources allow to fill the gaps in the data and allow for comparison at all. A detailed description of the indicators and what they capture is included in the Appendix B.

Whenever available, data was examined from 2005, 2010, 2015 and 2019, as the analysis aims to identify trends over the past 15 years. When data was not available for the indicated year, the data from the nearest available year was used (for example, 2016 instead of 2015). When no data was available for the whole of the UK, data from England was used, as it is the country of the UK with the largest population. Percentages were calculated where possible, using the corresponding populations to the indicator as a denominator, for example, LTC recipients in institutions as a percentage of the total population.

## Results

### Population statistics and health expenditure

**Fig. 1:**
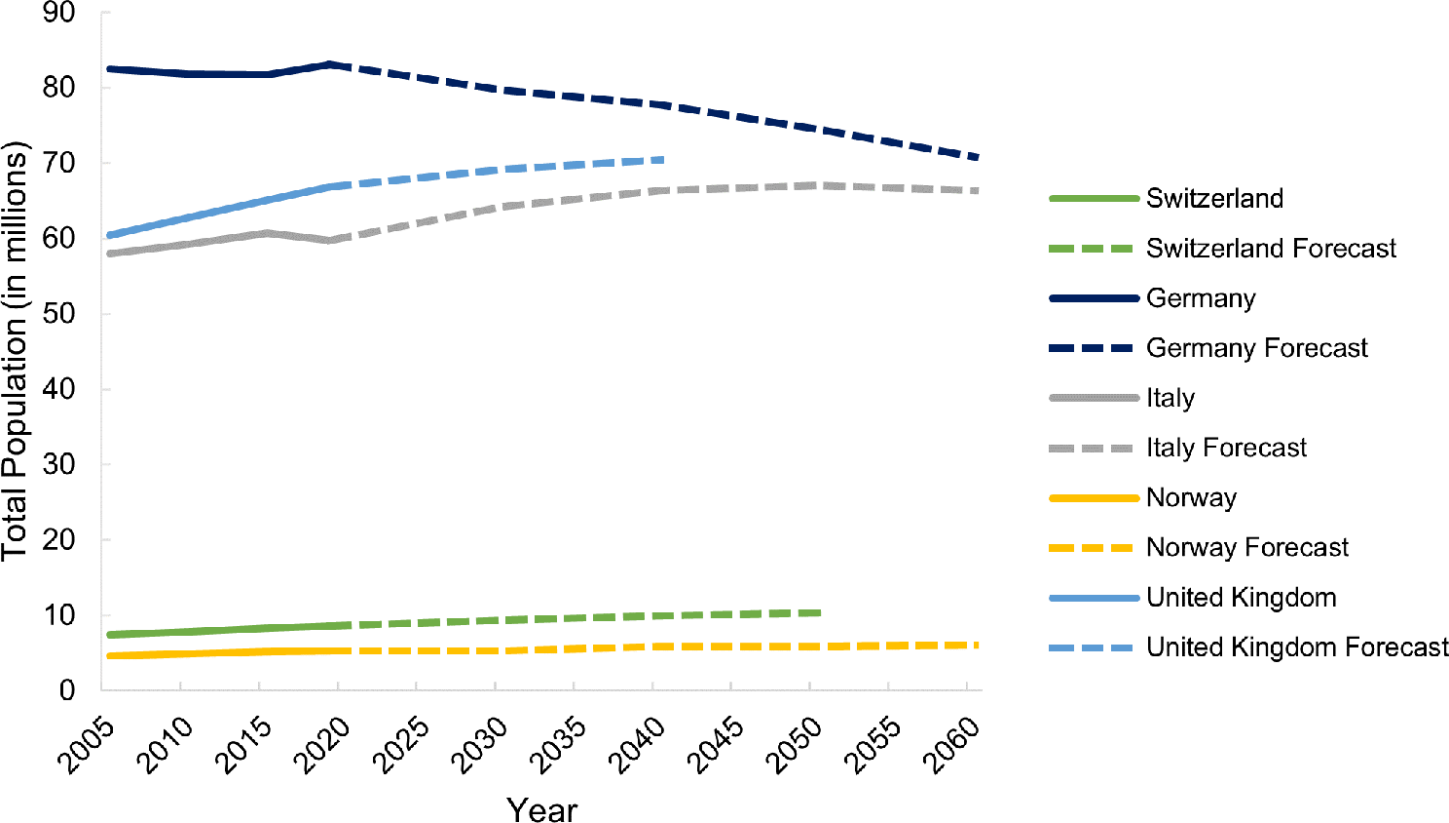
Past (2005–2019) and forecasted (2030–2060) total population of Switzerland, Germany, Italy, Norway and the United Kingdom

**Fig. 2:**
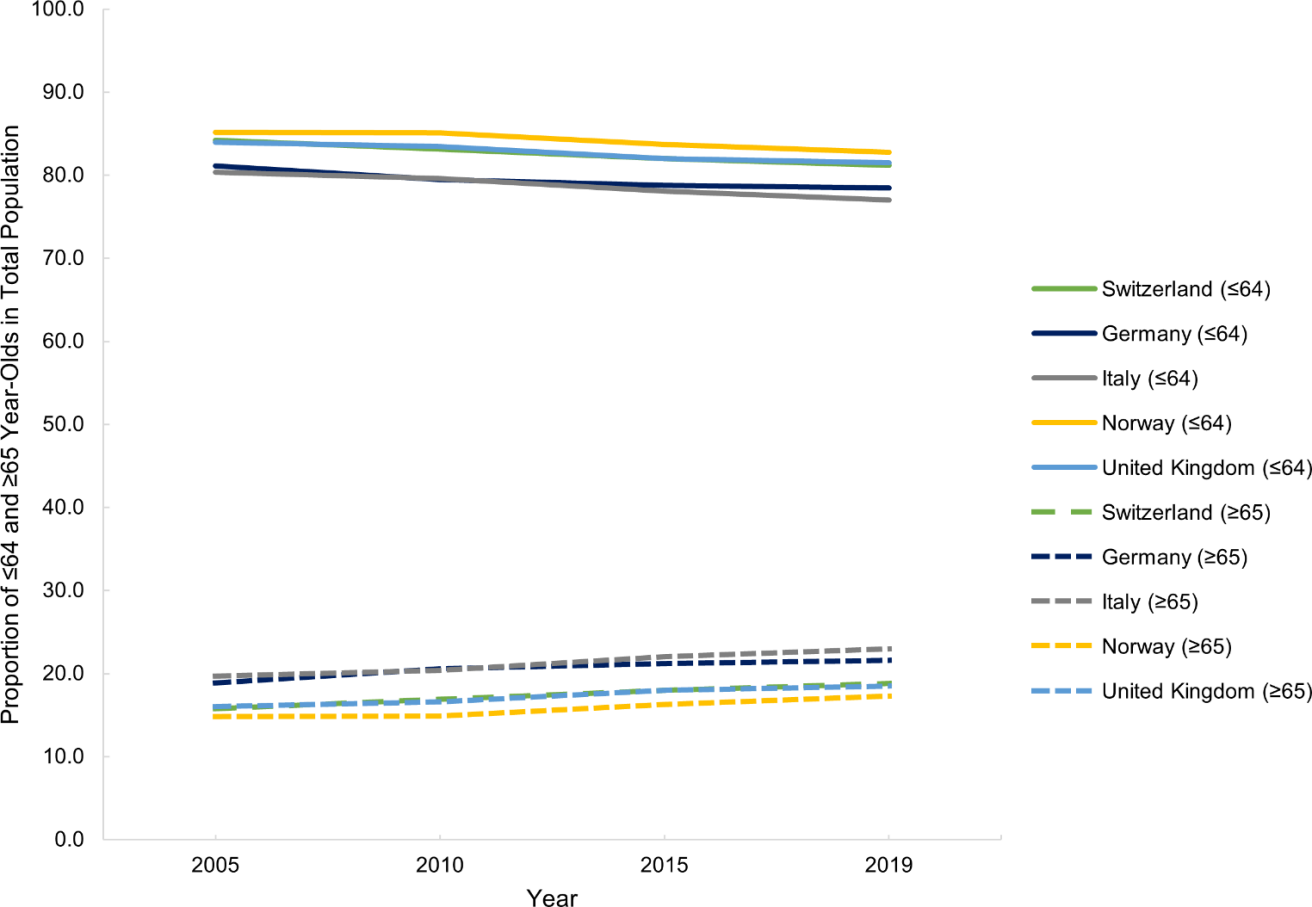
Proportion of ≤ 64 and ≥ 65 Year-Olds in Total Population, in Switzerland, Germany, Italy, Norway and the United Kingdom from 2005 and 2019

This section refers to Table S1 (all tables with detailed results are available in Appendix A). Switzerland, Norway and the UK’s populations have grown steadily from 2005 to 2019, whereas Italy’s population started to decline from 2015 onwards (Fig. 1). Germany had the largest population, and Norway had the smallest in 2019 (Fig. 1). The proportion of over 65-year-olds has grown in all five countries from 2005 to 2019 (Fig. 2). Italy has continuously been the population with the largest proportion of over 65-year-olds, increasing by 3.3% points from 2005 to 2019 (Fig. 2). Norway is the country with consistently the lowest proportion of over 65-year-olds, yet also increasing 2.5% points from 2005 to 2019 (Fig. 2).

**Fig. 3:**
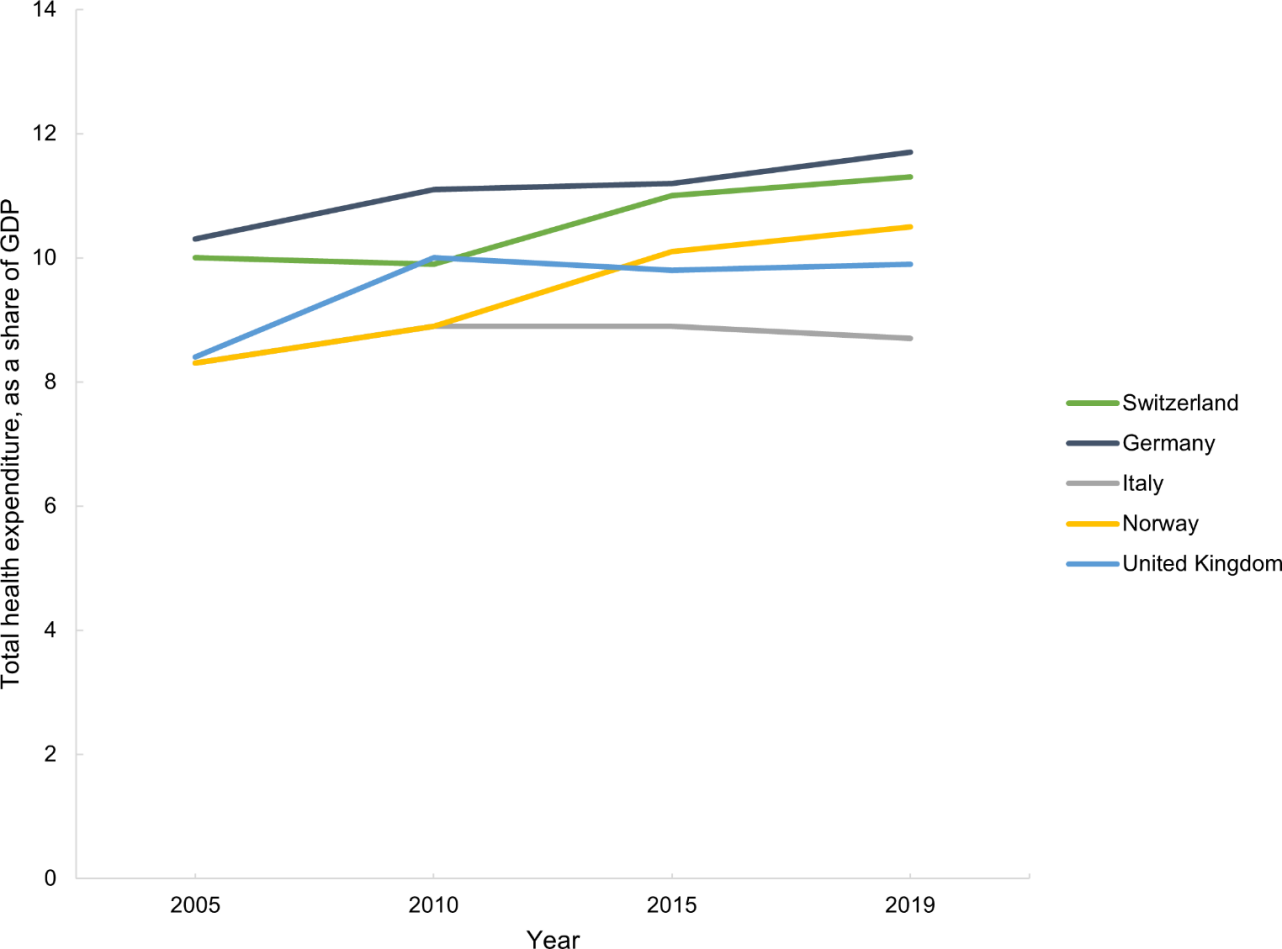
Total health expenditure, as a share of gross domestic product (GDP), in Switzerland, Germany, Italy, Norway and the United Kingdom from 2005 and 2019

Switzerland had the highest GDP per capita in 2019, followed by Norway, Germany, the UK and Italy (Table S1, Appendix A). The same pattern is consistent through each time point, although GDP per capita has increased in each country over the five time points (Table S1, Appendix A). The total health expenditure follows the same pattern as the GDP per capita. Switzerland has the highest total health expenditure per capita and Italy the lowest, spending approximately half of what Switzerland spends (Table S1, Appendix A). However, as a percent of GDP, the trend changes (Table S1, Appendix A). Germany had the highest total health expenditure, as a share of GDP, in 2019, and Italy the lowest (Table S1, Appendix A). The pattern of total health expenditure as a percent of GDP is consistent at all time points (Fig. 3).

### Need for long-term care

**Fig. 4:**
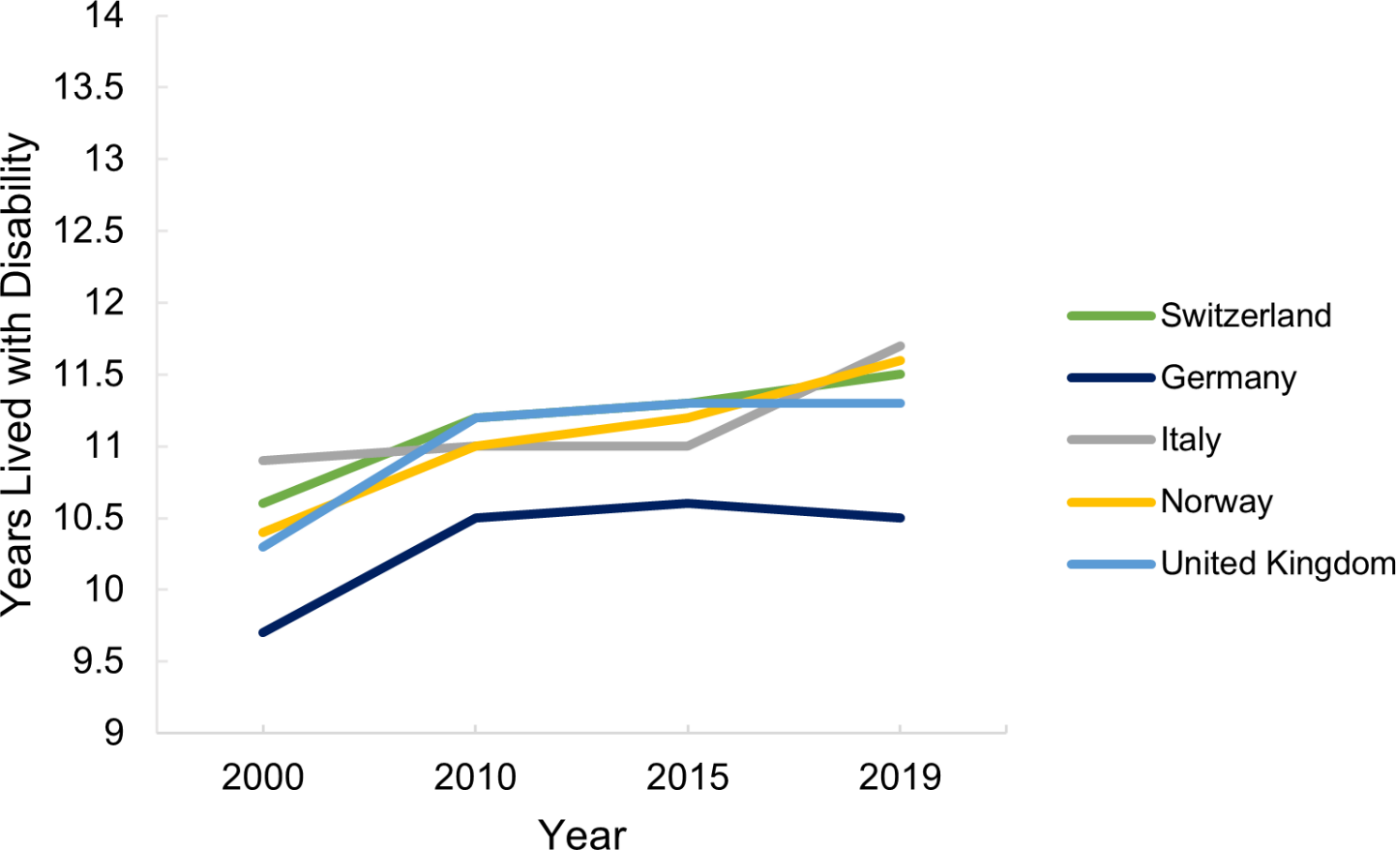
Years lived with disability in Switzerland, Germany, Italy, Norway and the United Kingdom from 2000 to 2019

This section refers to Table S2 (Appendix A). Across all time points, Switzerland had the highest life expectancy and healthy life expectancy at birth, with Italy close behind. The difference in years between life expectancy and healthy life expectancy is known as years lived with disability. As shown in Fig. 4, there is a trend of increasing years lived with disability in Switzerland, Norway and Italy. In Germany and the UK, the years lived with disability decreased from 2015 to 2019.

**Fig. 5:**
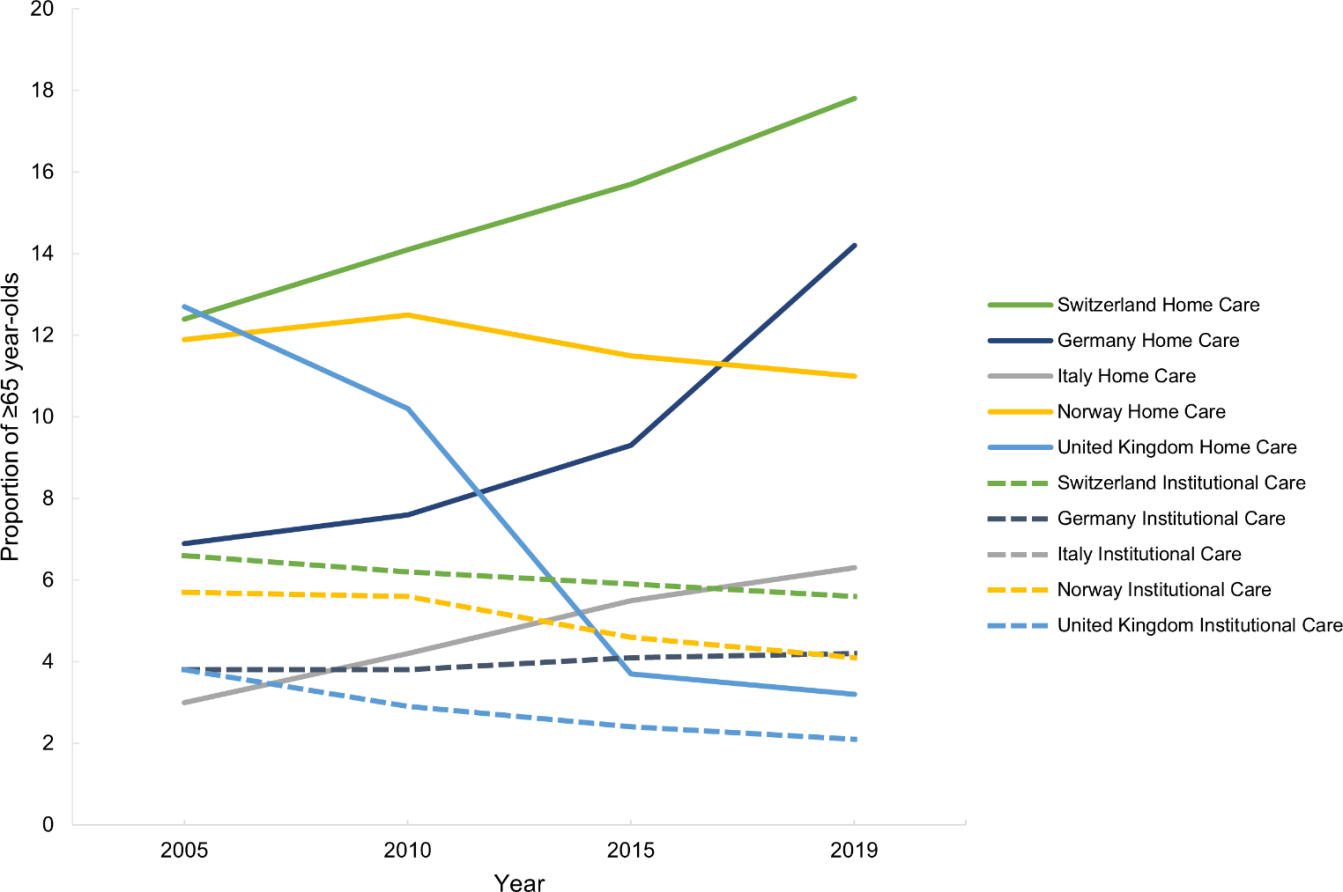
Proportion of ≥ 65 year-olds receiving long-term care in institutions and at home in Switzerland, Germany, Italy, Norway and the United Kingdom between 2005 and 2019

Switzerland has the highest proportion of over 65-year-olds receiving LTC both in institutions and at home (Fig. 5). In all countries, the proportion of over 65-year-olds receiving LTC in institutions has decreased from 2005 to 2019, except in Germany where this has increased (Fig. 5). In Switzerland, Germany and Italy the proportion of over 65-year-olds receiving LTC at home has increased since 2005, but the increase is more pronounced for Switzerland (Fig. 5). In Norway and the UK, this proportion has decreased (Fig. 5). Italian data was not available.

**Fig. 6:**
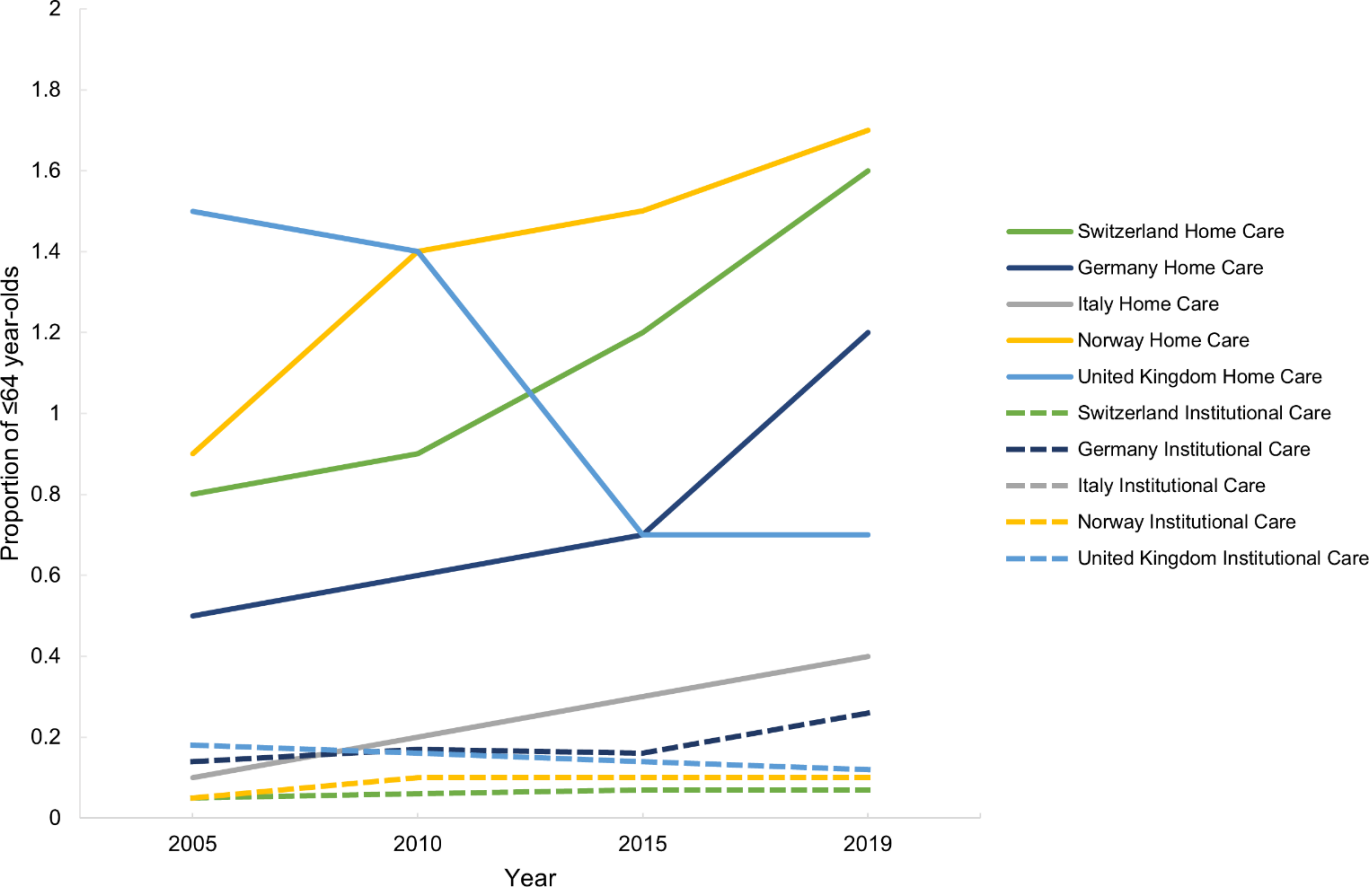
Proportion of ≤ 64 year-olds receiving long-term care in institutions and at home in Switzerland, Germany, Italy, Norway and the United Kingdom between 2005 and 2019

In all countries but the UK, the proportion of under-64-year-olds in LTC institutions has slightly increased over time, while the trend for the same group receiving care at home shows a more pronounced increase, especially in Switzerland, Germany and Norway (Fig. 6). In 2019, the countries with the highest proportion of this group in LTC at home were Switzerland and Norway (Fig. 6).

**Fig. 7:**
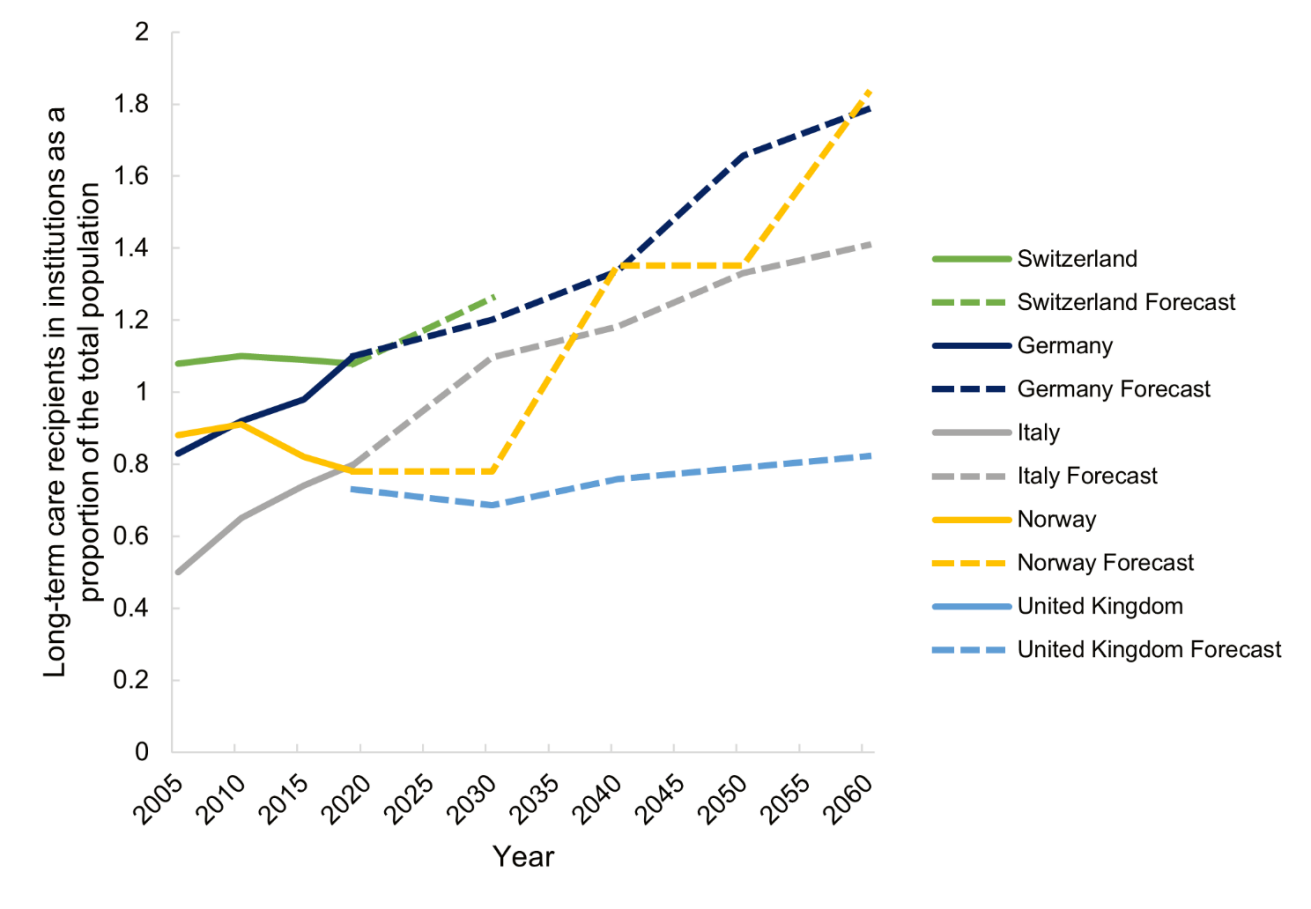
Past (2005–2019) and forecasted (2030–2060) long-term care recipients in institutions in Switzerland, Germany, Italy, Norway and the United Kingdom

**Fig. 8:**
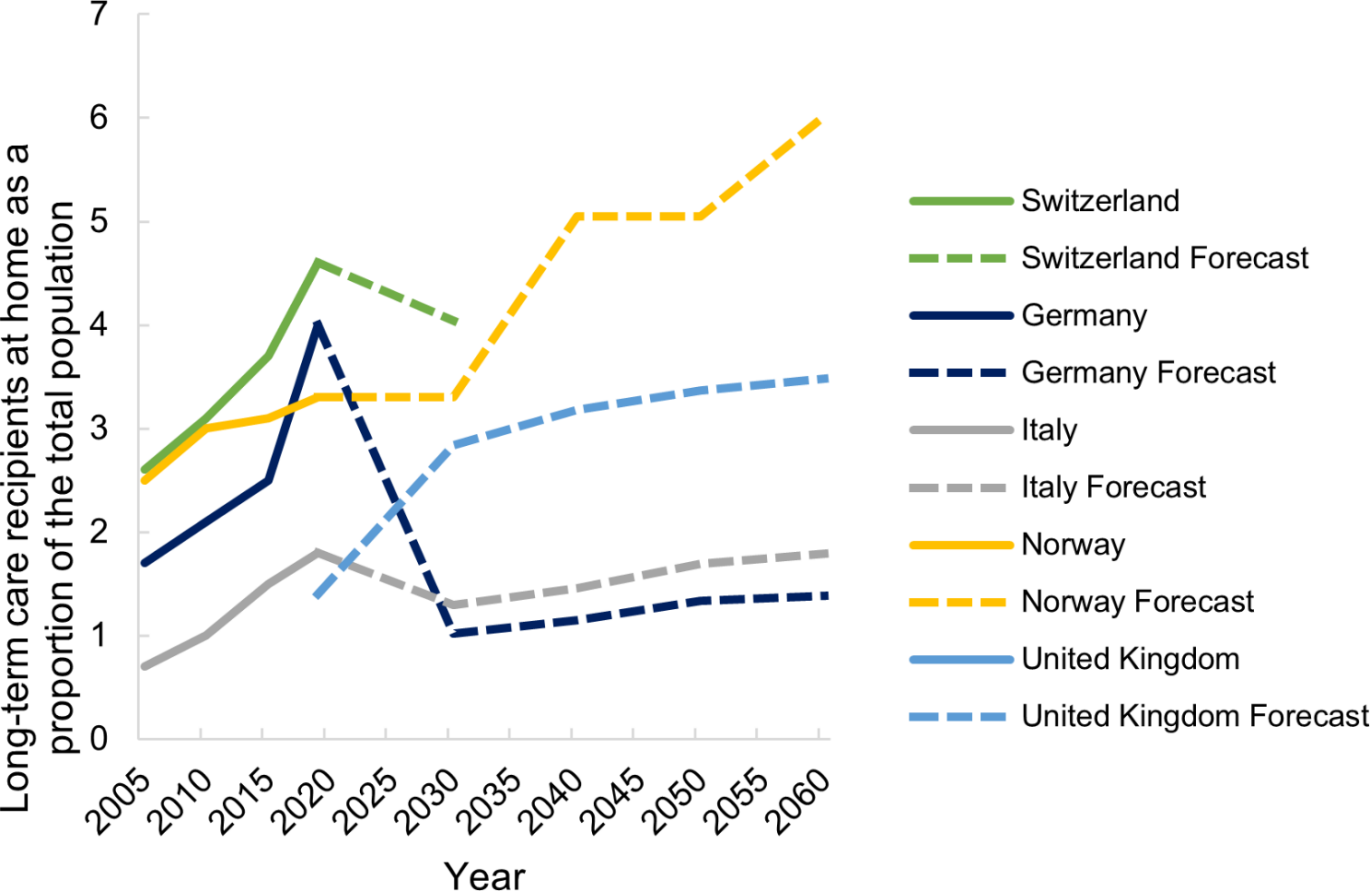
Past (2005–2019) and forecasted (2030–2060) long-term care recipients at home in Switzerland, Germany, Italy, Norway and the United Kingdom

As of 2030, the number and proportion of persons receiving LTC in both institutions and at home is predicted to increase in all countries (Figs. 7 and 8). This increase is also projected for Switzerland but no predictions past 2030 are available so far.

### Long-term care financing

This section refers to Table S3 (Appendix A). Total expenditure on health-related LTC as a share of GDP was consistently highest in Norway (3.1%, 2019) (Table S3, Appendix A). In Switzerland and Germany total expenditure on health-related LTC as a share of GDP was slightly above 2% in 2019, slightly below 2% in the UK and below 1% in Italy (Table S3, Appendix A). In Switzerland, Germany and Norway, the total expenditure on health-related LTC as a share of GDP has increased over time while it remained stable in Italy and the UK (Table S3, Appendix A). The trends are the same for total expenditure on health-related LTC as a share of health expenditure as well as for total expenditure per capita (Table S3, Appendix A).

Public expenditure on health-related LTC as a percent of GDP is projected to increase in all five countries (Fig. 7). According to the European Commission’s 2021 Ageing Report, the highest increase will take place in Norway (2.5% points) between 2030 and 2060, followed by a 1.2% point increase in Switzerland (Table S3, Appendix A). The UK and Italy are projected to have the smallest increase (0.2% points) (Fig. 7).

**Fig. 9:**
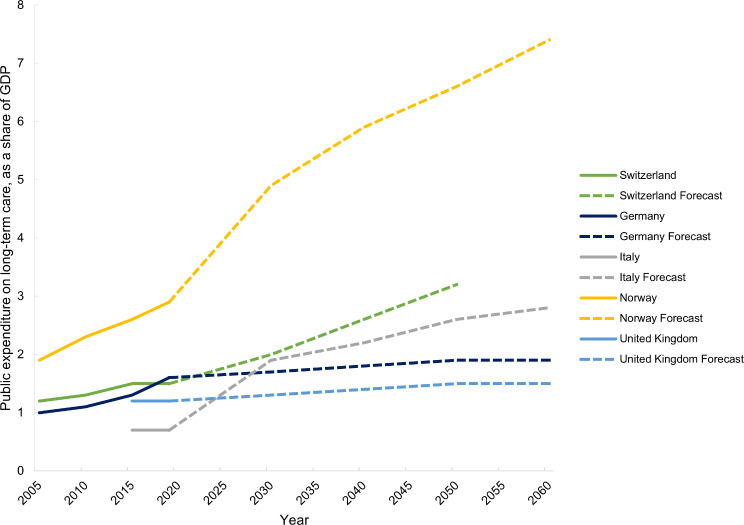
Past (2005–2019) and forecasted (2030–2060) public expenditure on long-term care as a share of gross domestic product (GDP) in Switzerland, Germany, Italy, Norway and the United Kingdom

Norway’s public expenditure on health-related LTC has been the highest and Italy’s the lowest across all time points (Fig. 9). As a share of health expenditure, Norway’s public expenditure on health-related LTC was twice as much as in Switzerland and Germany (Table S3, Appendix A). Public expenditure on health-related LTC as a share of GDP increased by 1.0% points from 2005 to 2019 in Norway and 0.6% points in Germany, but only 0.3% points in Switzerland (Table S3, Appendix A).

**Fig. 10:**
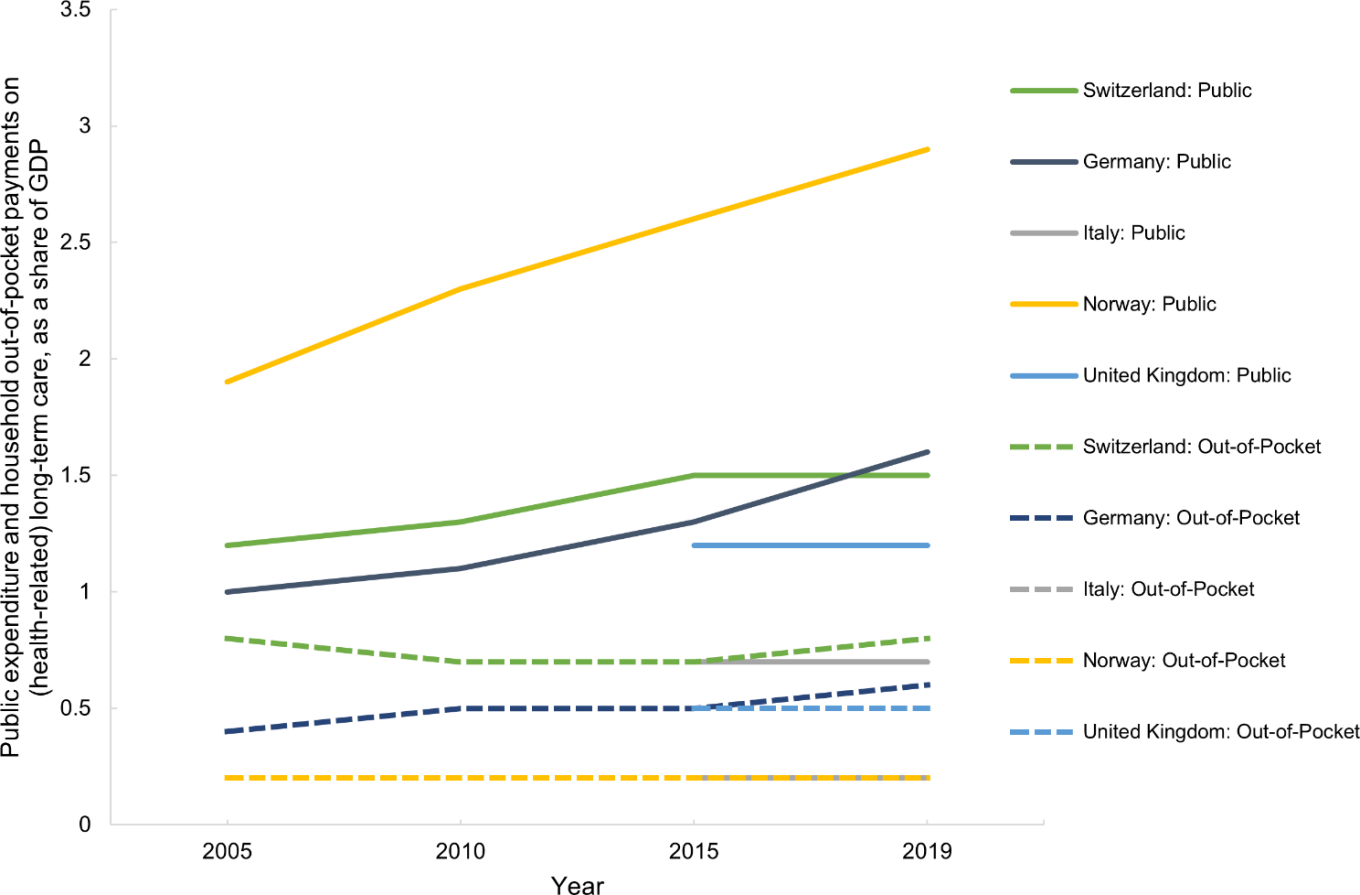
Public expenditure and household out-of-pocket payments on (health-related) long-term care, as a share of GDP, in Switzerland, Germany, Italy, Norway and the United Kingdom between 2005 and 2019

Household out-of-pocket payments on health-related LTC are highest in Switzerland at ca. 7% of total health expenditure in 2019, followed by just above 5% in the UK and 5% in Germany, and the lowest in Italy and Norway (Table S3, Appendix A). In Germany and the UK, household out-of-pocket expenditure on health-related LTC as a share of health expenditure has been increasing while in Norway and Switzerland, this trend is decreasing (Table S3, Appendix A). Similar trends can be seen in household out-of-pocket payments on health-related LTC as a share of GDP, as seen in Fig. 10.

#### Long-term care service delivery

**Fig. 11:**
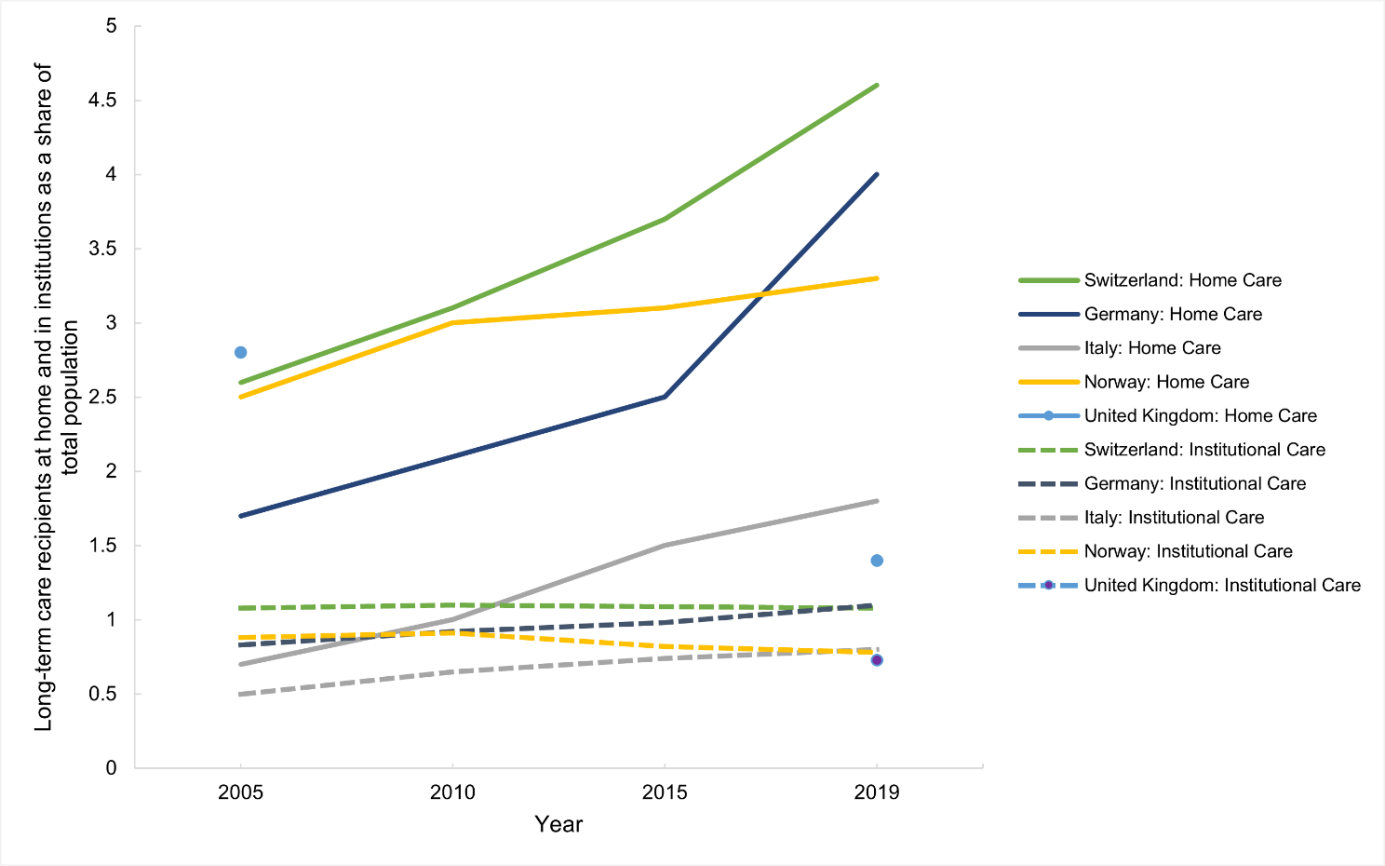
Long-term care recipients at home and in institutions, as a share of the total population, in Switzerland, Germany, Italy, Norway and the United Kingdom between 2005 and 2019

This section refers to Table S4 (Appendix A). Germany and Switzerland had the highest and the UK had the lowest number of LTC recipients in institutions as a percentage of the total population in 2019 (Fig. 11). Over time, this percentage has stayed relatively stable in Switzerland but has increased in Germany and Italy by around 0.3% points from 2005 to 2019 (Table S4, Appendix A). In Norway, the same percentage increased slightly from 2005 to 2010 and then decreased to below the 2005 value in 2019 (Fig. 11).

The country with the highest number of LTC recipients at home, as a percentage of the total population, is also Switzerland, while the countries with the lowest number are Italy and the UK (Fig. 11). In all countries except the UK, this number has increased since 2005 (Fig. 11).

**Fig. 12:**
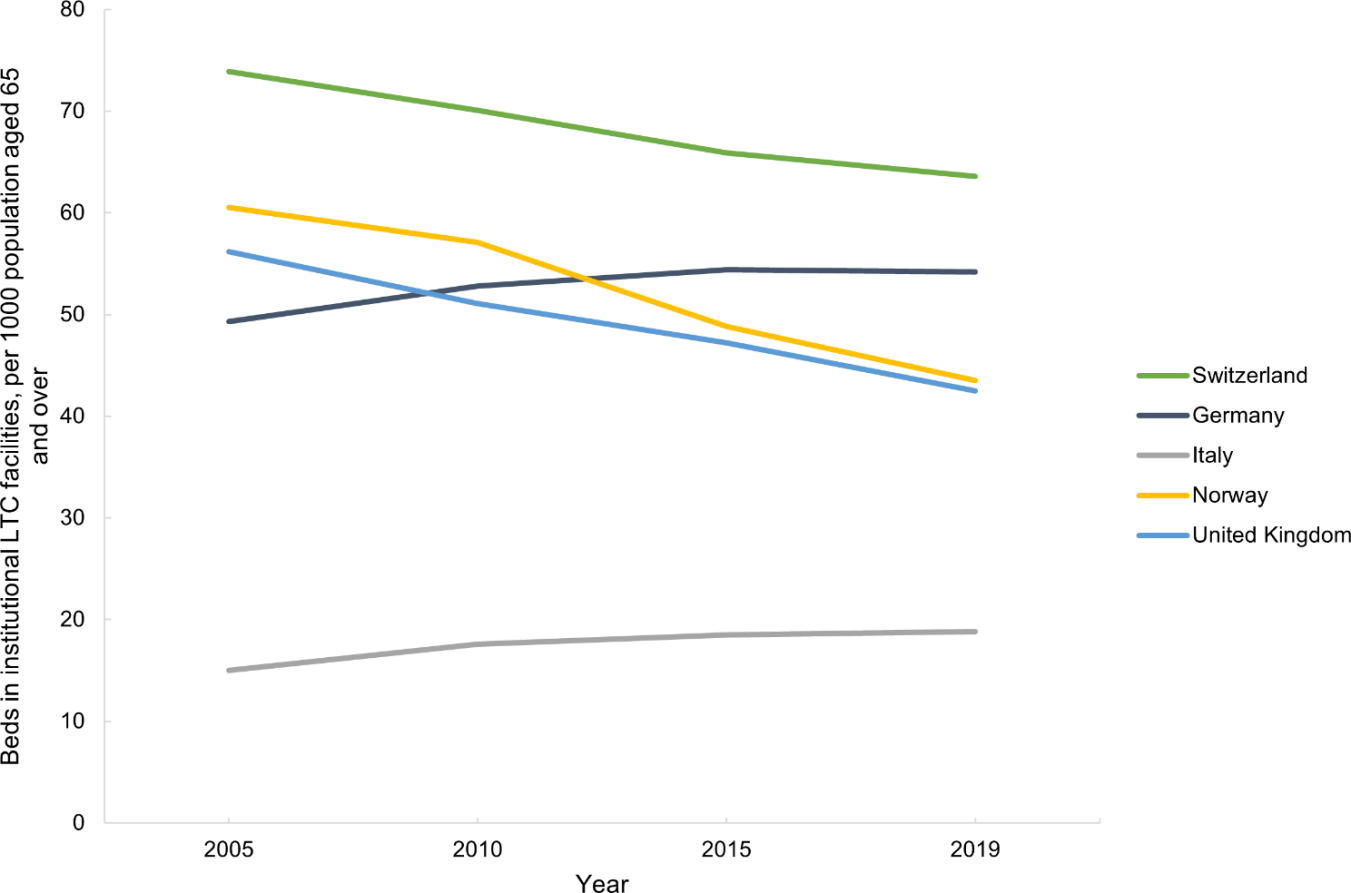
Beds in institutional LTC facilities, per 1000 population aged 65 and over, in Switzerland, Germany, Italy, Norway and the United Kingdom between 2005 and 2019

Switzerland has the highest and Italy the lowest number of beds in institutional LTC facilities per 1000 population aged 65 and over (Fig. 12). Although the absolute number of beds in Switzerland has increased, the number of beds as a percentage of the population aged 65 and over, has decreased with time (Table S4, Appendix A). In Norway and the UK, the absolute number of beds has decreased over time (Table S4, Appendix A). In Germany and Italy, the absolute number of beds and number of beds per 1000 population aged 65 and over has increased since 2005 (Table S4, Appendix A).

### Long-term care workforce

**Fig. 13:**
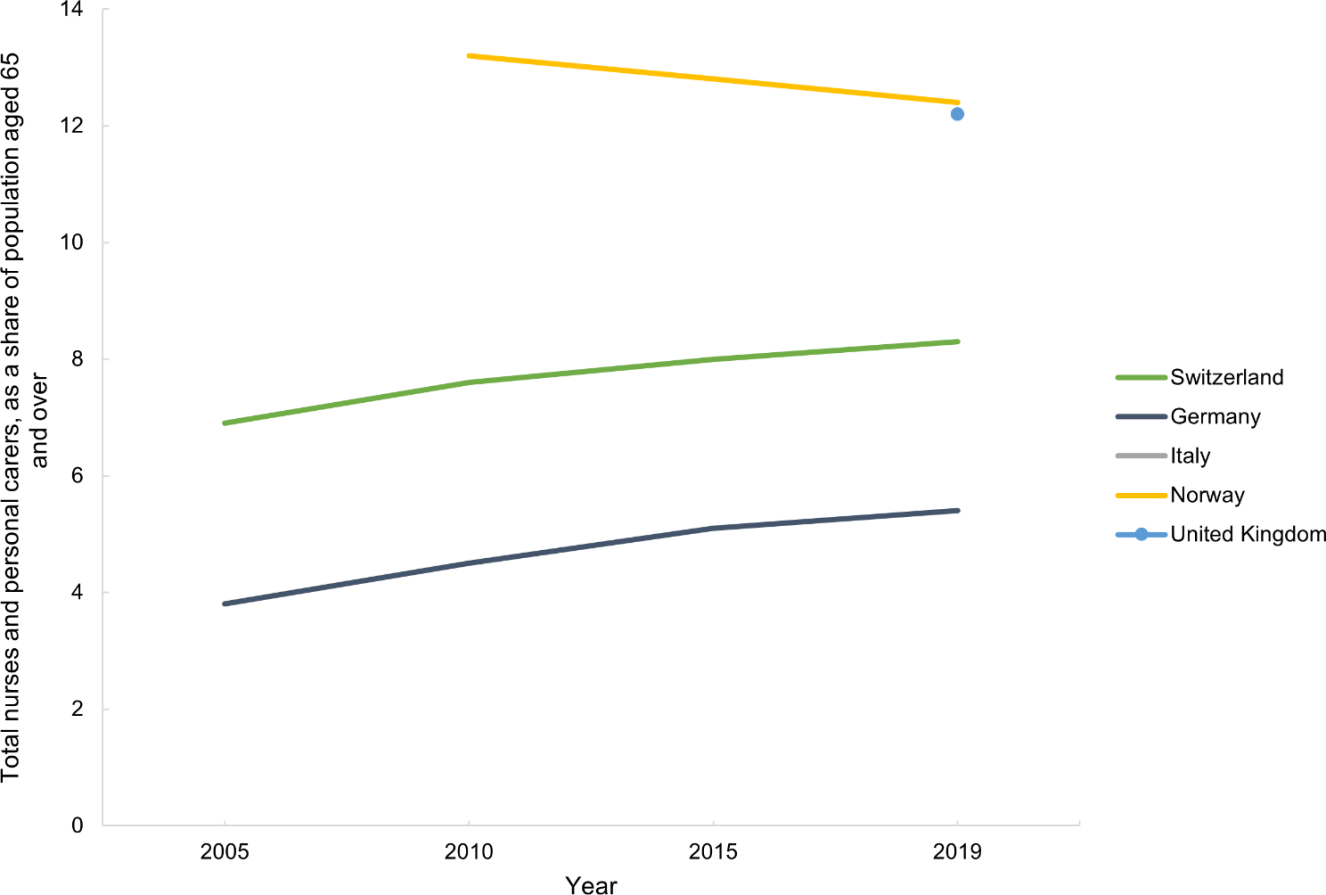
Total nurses and personal carers, as a share of population aged 65 and over, in Switzerland, Germany, Italy, Norway and the United Kingdom between 2005 and 2019

This section refers to Table S5 (Apprendix A). In Switzerland and Germanythe percentage of total nurses and personal carers relative to the population aged 65 and over has increased over time (Fig. 13). Norway and the UK have the highest proportion of nurses and personal carers, followed by Switzerland (Fig. 13). The UK lacks data previous to 2020 and Italy lacks data for all time points.

**Fig. 14:**
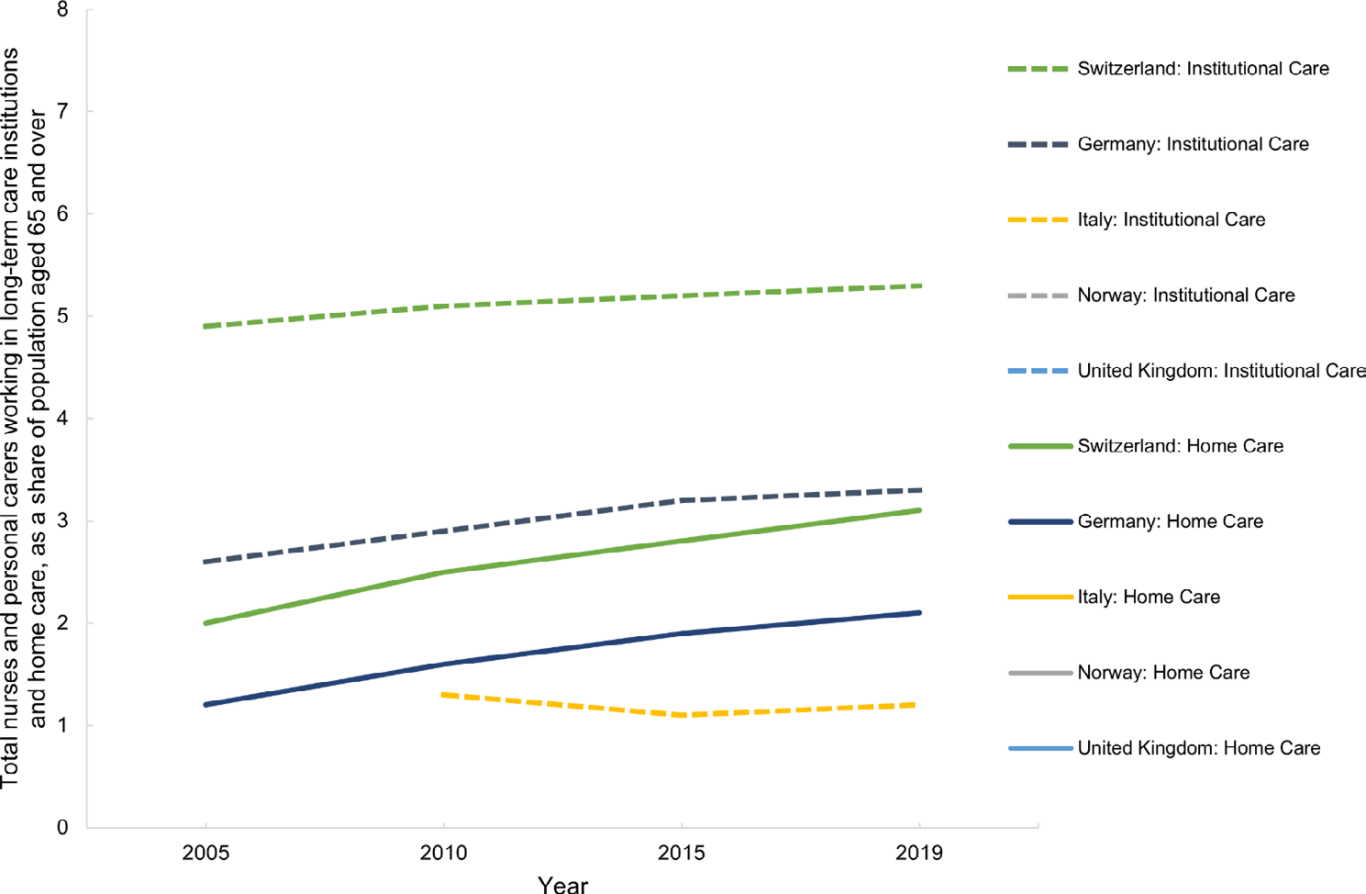
Total nurses and personal carers working in long-term care institutions and home care, as a share of population aged 65 and over, in Switzerland, Germany, Italy, Norway and the United Kingdom between 2005 and 2019

Switzerland, Germany and Italy have a higher proportion of nurses and carers working in LTC institutions than in long-term home care (Norway does not have available data) (Fig. 14). In Germany and Switzerland, both the total and the relative number of nurses and carers as a proportion of the population aged 65 and over in both institutions and home care have increased since 2010 (Table S5, Appendix A).

**Fig. 15:**
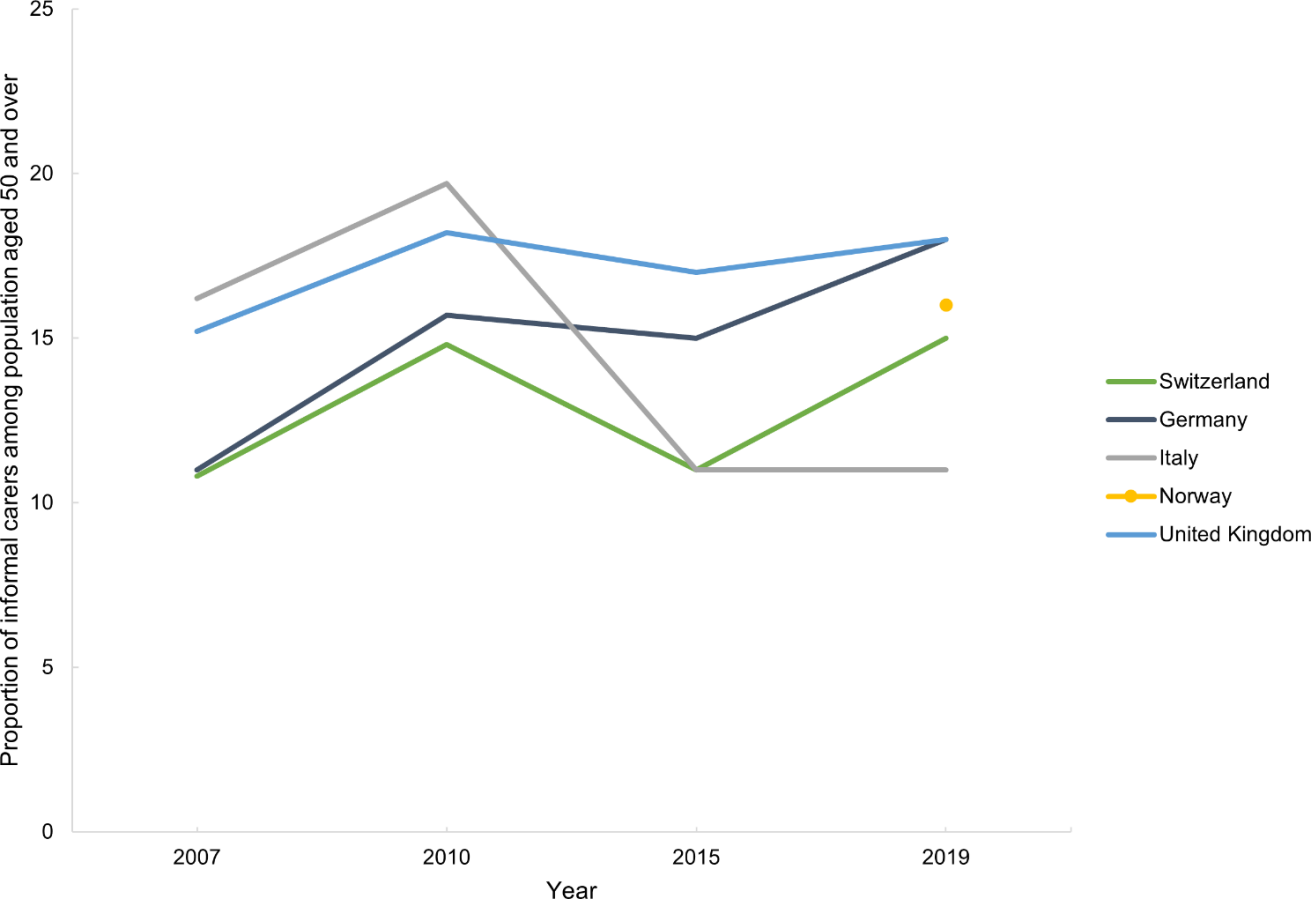
Proportion of informal carers among population aged 50 and over, in Switzerland, Germany, Italy, Norway and the United Kingdom between 2007 and 2019

The proportion of informal carers among the population aged 50 and over has increased in Switzerland, Germany and the UK, and decreased in Italy since 2007 (Fig. 15). Norway does not have data available for this indicator, but the percentage of unpaid care workers among persons 16 years and over was 16% in 2019 (Table S5, Appendix A).

Table 3 presents an overview of the main results corresponding to each study aim.

**Table 3:** Study aims and summary of main results

Aim	Main reresults
**Trends over the past 15 years in need for LTC**	• Although Switzerland has the highest life expectancy and healthy life expectancy at birth, there is a trend of increasing years lived with disability in Switzerland.
	• Switzerland has the highest proportion of over 65-year-olds receiving LTC both in institutions and at home and the proportion of recipients is increasing in both.
	• Switzerland has had the most pronounced increase in the proportion of over 65-year-olds receiving LTC at home.
**Trends over the past 15 years in LTC financing**	• Switzerland’s total expenditure on LTC is below Norway’s and similar to Germany’s, and is increasing.
	• Public expenditure on LTC has increased less in Switzerland than in Norway and Germany.
	• Household out-of-pocket payments on health-related LTC are highest in Switzerland.
**Trends over the past 15 years in LTC service delivery**	• Germany and Switzerland had the highest number of LTC recipients in institutions as a percentage of the total population in 2019. Over time, this percentage has stayed relatively stable in Switzerland.
	• Switzerland has the highest number of beds in institutional LTC per 1000 population aged 65 and over. Although the absolute number of beds in Switzerland has increased, the number of beds as a percentage of the population aged 65 and over has decreased with time.
	• Switzerland also has the highest number of LTC recipients at home, as a percentage of the total population.
**Trends over the past 15 years in LTC workforce**	• In Switzerland, the percentage of total nurses and personal carers relative to the population aged 65 and over has increased over time.
	• Norway and the UK have the highest proportion of nurses and personal carers, followed by Switzerland.
	• Switzerland has a higher proportion of nurses and carers working in LTC institutions than in long-term home care.
	• In Switzerland, both the total and the relative number of nurses and carers as a proportion of the population aged 65 and over in both institutions and home care have increased since 2010.

*Abbreviations* LTC (Long-Term Care)

## Discussion

In line with a European trend, Switzerland’s population of 65-year-olds and over is increasing, along with the number of years lived with disability, leading to a significant increase in the need for LTC. This study uses a comprehensive set of international and national indicators to compare LTC financing, service delivery and workforce in Switzerland to Germany, Italy, Norway and the UK in order to inform policy-making. Due to varying data sources, the results must of course be interpreted with caution. After Norway, Switzerland has the highest LTC expenditure as a share of the GDP. In comparison, Switzerland has the highest out-of-pocket payments for health-related LTC. In all countries, there are more recipients of LTC at home than in institutions. However, like in Germany and Italy, Switzerland still has more LTC workforce in institutions than at home. In comparison to Germany and the UK, Switzerland has a lower number of informal carers as a proportion of 50-year-olds and over, as well as fewer nationally available services for informal carers compared to Germany, Italy, Norway and the UK.

### Need for LTC

The results portray a very slight expansion of morbidity between the years 2000 and 2019, between 0.8 and 1.2 increase in years lived with disability over a 19-year-period in the five compared countries. In the year 2000, Fries argued that while healthcare improves and life expectancy increases, humans should also expect to live a life that is less afflicted by disability [28]. This predicted reduction of years lived with disability has been named compression of morbidity. Chatterji et al., however, highlighted the importance of data availability and quality in this debate and suggested that there is conflicting evidence [29]. The years lived with disability in this case was calculated using life expectancy at birth and subtracting healthy life expectancy at birth [3]. As Chatterji et al. suggest, the available literature paints a conflicting image. A Norwegian study using Global Burden of Disease data to assess morbidity across Norwegian regions has concluded that Norway has a stable level of life expectancy, healthy life expectancy, years lived with disability and disability-adjusted life years across all regions [30]. This suggests that Norway’s political push to reach equality in access and availability of healthcare services across regions has been successful on this front. In our results, Germany was shown to have about 1 year of years lived with disability less than the other countries across all time points, while also slightly increasing over time. Contrarily, a study conducted by Trachte, Sperlich and Geyer showed a compression of morbidity in the older population using subjective and functional health data from the German Socio-Economic Panel [31]. However, Tetzlaff et al. demonstrated an expansion of morbidity in the region of Lower Saxony based on data from health insurance claims [32]. Remund et al. suggest that there is a divergence of morbid years by educational levels in Switzerland, which may be associated with the high out-of-pocket costs of preventive care [33]. This study highlights the important differences in morbidity trends based on sex and education level, a depth that our results do not reach. Understanding the developing needs of a population is of utmost importance to properly plan services accordingly. The evidence for trends in morbidity development is inconclusive and therefore it would be important for Switzerland to delve deeper into the data available to understand how methodological and regional differences may affect LTC planning.

### LTC Financing

A large concern of the Swiss government is optimizing the funding of LTC [13, 18]. The results of our study showed that Switzerland’s public expenditure on LTC, as a share of GDP, is below Norway’s and similar to Germany’s, but the out-of-pocket expenditure of households exceeds that of all other countries. It has been predicted that if the Swiss financing system for LTC remains unchanged, the costs of LTC will surpass the available level of federal and cantonal funds by 2030 [18]. Other concerns of the Swiss Federal Council, as expressed in the report ‘Review of the current situation and prospects in the field of LTC’ of the Swiss Federal Council from May 2016, are the rising out-of-pocket expenditure and health insurance premiums [18]. In the same report, several options for future financing of the LTC system were suggested. One financing option presented was the implementation of a LTC insurance. An example of such a financing system can be seen in Germany. In 1995, Germany opted to implement a mandatory LTC insurance, as Germany faced similar challenges as Switzerland today [34]. The LTC insurance in Germany has removed a significant burden from both the German government and individuals with LTC needs [35]. A LTC insurance would target other goals of the Swiss Federal Council as well, including being within the scope of the constitution and the ability to cover a larger number of services than currently covered [18]. Such services include help with cleaning, cooking, and transportation [18]. To redefine services and coordinate between health insurances, cantons, state and a LTC insurance, additional administrative resources may be necessary, however, these efforts may benefit the financial sustainability of the LTC system in the long run.

### LTC Service delivery

In Switzerland, compared to the other countries, both institutional and home care services are highly used. Yet, the demand for home care services is increasing while institutional services remain stable. On a national level, Switzerland has pushed the LTC strategy “home care before institutional care” (translated from *Ambulant vor Stationär*). However, as service delivery is the responsibility of cantons, the services available to residents vary highly between cantons. The Swiss Health Observatory demonstrated that three categories of care models exist across Swiss cantons [36]. Central Switzerland, including cantons Lucerne and Schwyz, relies on institutional care whereas western Switzerland, including French speaking cantons Vaud and Geneva, rely mostly on home care. The third category of cantons, such as cantons Zurich, Bern and Grisons, use a mixed-model. There has also been a push for the implementation of more services, apart from traditional home care and institutional care services, such as day and night care, independent living apartments, and assisted living [18]. In 2019, there were just under 32’000 independent living apartments, around 3’000 day care spots and 350 night care spots available in Switzerland. Another push towards increasing the diversity and availability of home care services develops from the Convention on the Rights of Persons with Disabilities’ (CRPD) Article 19, which states that persons with disabilities have the equal right to living independently and being included in the community [37]. This article expresses the need for ensuring a plethora of services available to choose from to meet the needs of persons with disabilities and persons with LTC needs. It is important to continue to push for the development of home care and intermediate forms of LTC that promote independent and community living.

### LTC Workforce

It has been acknowledged by the Swiss Federal Council that informal care is an irreplaceable, cost-effective form of care, and therefore relief for informal carers was named one of the 7 concrete action areas for improving LTC [18]. One of the benefits of informal carers is that they are most often trusted and valued members of the patient’s environment [38]. They contribute a significant part of care in Switzerland, especially when professional home care services are unavailable or where geographical inequalities exist [39]. According to Rudnytskyi and Wagner, informal care is both complementary and substitutional to professional care in Switzerland [40]. In 2020, a federal law was passed to increase the support of informal caregivers in Switzerland, in response to a 2014 strategic report on support for caring relatives from the Swiss Federal Council, implementing paid leave as well as insurance supplements and credits for informal carers. However, in comparison to other countries, Switzerland does not have direct financial support for caregivers on a federal level. In a report published by Eurohealth in 2019, it was suggested that cash benefits and home care care services improve personalized care and personal satisfaction with care, using England and Germany as examples [41]. A recent study from Sweden also suggested that services that support informal caregivers could be cost-effective [42]. Implementing cash benefits for informal carers nationally could relieve burden from both professional and informal carers, increase personalization and care satisfaction in addition to increasing the cost-effectiveness of care.

Ensuring sufficient workforce to support the LTC needs of the population is a key component of Switzerland’s Health2030 strategy [13]. The increasing years lived with disability as well as the increasing number of recipients of LTC within all age groups shows the increasing need for LTC. Therefore, an equivalent increase in workforce is needed. In Switzerland, the topic of an insufficient nursing workforce is very prevalent. Not only as part of the Health2030 strategy, but also in November 2021, a federal popular initiative was voted on and passed that aims to improve support and working conditions for nurses and build capacity in the nursing workforce [43]. This topic is also highly relevant in other OECD countries [44]. It is however necessary to understand how to efficiently distribute the workforce for an effective and sustainable system. From our results, the trend towards more care at home is visible in the service delivery of all countries. However, in all countries other than the UK, there remains more workforce in institutional LTC than in home care. Although four times more persons receive care at home in Switzerland, over 60% of its nursing and caring workforce worked in LTC institutions in 2019. Additionally, the expenditure of LTC institutions was six times higher than that of home care services in 2020 in Switzerland [45]. Increasing the amount of nurses will not be enough. When it comes to the planning of personnel and financial resource allocation, there is good reason to prioritize home care.

From the indicators under the aim ‘need for LTC’ we can understand the demand for LTC, while the indicators under ‘LTC service delivery’ and ‘LTC workforce’ tend toward understanding the supply of services. From the trends and the discussions above we can observe that demand for LTC in Switzerland is increasing overall and shifting towards home care, however, supply is still heavily involved in institutional care, given by the number of beds and workforce available and still increasing. In comparison, in the UK, the years lived with disability remained stable between 2010 and 2019. However, we see a drop in the supply of LTC between 2010 and 2015. It is known that disability figures are conditional on legal definitions and the approach used for disability assessment [46]. The drop in supply is therefore likely due to the introduction of The Care Act in 2014, as it introduced new eligibility criteria and means-testing. These criteria have been criticized to restrict the access of persons with LTC needs to LTC services [47]. Although supply may be limited, the UK has succeeded in shifting supply from institutional to home care by reducing beds in institutional LTC facilities and increasing staff in home care.

### Data quality and availability

In order to improve LTC systems, monitoring the current state is crucial [48]. Our results, similar to a recent European Commission publication [48], show that all countries have gaps in data availability and gaps in data quality. This is visible in the number of gaps in the data as well as the variety of sources used to fill the gaps in the data in this report. Additionally, due to the lack of data availability, this report is restricted to LTC with a focus on chronological age and only health-related expenditure on LTC. Also shown in our results was that some predictions have failed to account for the shift in service preference and the speed of this shift. Switzerland has fewer gaps in data presented in this paper than other countries, such as Italy and the UK. However, Switzerland has fewer available predictions. An expansion of the data collected and published as well as predictions made on LTC by national statistics offices would allow for better strategic planning of services, workforce and financing.

### European care strategy

This paper covers similar issues to the recently published European Care Strategy, with a focus on the improvement of affordable LTC, supporting the movement towards home care services, improving the support given to both the professional workforce and informal carers, and improving the amount and quality of LTC data [49]. What was not covered in this report, but is a focus of the European Care Strategy, is improving quality in LTC [49]. One of the aims of the European Care Strategy is to improve quality within LTC by developing a quality framework for all LTC services [49]. Quality standards should be applicable to all LTC providers [49]. Assurance mechanisms to ensure quality are equally important [49]. Switzerland has a national quality framework for institutional LTC [50], but not for community care and informal care [51]. Although Switzerland is not a member state of the European Union, this European movement will stimulate innovation and affect LTC systems in countries surrounding Switzerland. It is important for Switzerland to continue to monitor, learn and use the momentum to stimulate positive change locally.

All compared countries follow a medical model to assess eligibility for LTC services, as seen in Table 1, involving a general practitioner or medical commission in the prescription of care. The UN CRPD and EU recommendations emphasize that clear, objective criteria that also take social and non-medical factors into account should be used in the assessment of LTC needs [37, 49]. In Switzerland, both service eligibility and financial access are determined by medical criteria. Germany is the only country in this comparison that has a separate LTC insurance, which has enabled a better integration of informal care and non-health-related LTC activities into the LTC system [52]. The WHO’s definition of LTC encompasses a broad range of health and social services, including cooking, cleaning, and supervision, indicating that LTC services should go beyond medical services. Thus, the eligibility criteria should reflect this broader scope.

### Limitations of study

Cross-country comparison is a method used to learn about national policies and systems and to learn lessons from the successes and failures of the systems and policies of other countries [53]. This analysis was able to paint a broad picture of LTC in the 5 compared countries. Nonetheless, this method comes with limitations that should not be ignored. Indicators were selected from sources such as the World Bank and OECD, which are often used for country comparisons. Any missing data was selected and added from other reliable international and national sources. The indicators were selected as the most informative yet complete and comparable between the selected countries. However, the true comparability of the indicators is often limited due to differences in data collection between countries. For example, for the indicator of LTC recipients at home, persons who only receive assistance with instrumental activities of daily living are excluded in Germany. In Switzerland, the OECD reports that “a small minority of recipients may only get help for instrumental activities of daily living” [54]. The other countries have not reported on this aspect. Additionally, some indicators that would have been beneficial for the analysis are missing from the sources used. For example, the proportion of LTC recipients receiving informal care as well as a measurement of unmet LTC needs. Future work may extend the list of indicators presented in this paper with data from SHARE [55] and ELSA [56].

Trends over time were compared between the five countries. As populations are ever-changing, policies and systems must continue to change with them. In order to compare countries with different demographics, proportions were used as a quantitative measure for comparison. However, with populations changing differently in each country from year to year, the differences in the percent change may be significantly impacted. The absolute numbers for indicators were presented alongside the proportions where possible, in order to give a complete understanding of the data.

This paper compared Switzerland to four other European countries. National data was presented in order to compare countries’ LTC systems. However, regional differences and thus regional inequalities within countries were not highlighted. As presented in the results, LTC is often organized at a regional level, and therefore further research should aim to include these regional differences within comparisons. Countries’ management of regional differences is as much of interest as national-level policies.

LTC is a multifaceted service that is provided within different areas of policy. Due to data quality and availability, the scope of this analysis was limited to the elderly population and the health-related expenditure of LTC. In the future, data should aim to cover all areas described by the given WHO definition of LTC, including all ages and all areas of the LTC system.

## Conclusion

This analysis adds to the topical discussion on LTC policy and LTC systems in Switzerland and Europe, by connecting relevant policy discussions to trends in need, financing, workforce and service delivery in LTC. The structure of LTC systems varies widely from country to country. However, in all countries, LTC policy must be multifaceted to meet different aims, including public and private expenditure, formal and informal workforce, availability and delivery of services and quality assurance [57]. Switzerland’s LTC system, in comparison to Germany, Italy, Norway and the UK, has high out-of-pocket expenditure, a high proportion of recipients of both institutional and home care, a high proportion of beds in institutional LTC, and low support for informal carers. It is therefore important to improve the affordability of LTC, continue to support the shift from institutional towards home care services, improve the support given to both the professional workforce and informal carers, and improve the amount and quality of LTC data in Switzerland. Similarly to other European countries, Switzerland has high regional variation in the availability and accessibility of LTC services. This variation was not covered in this analysis. Future studies may aim to map the regional variation of LTC policies in order to understand the gaps in the LTC system more accurately.

### Electronic supplementary material

Below is the link to the electronic supplementary material.

Supplementary Material 1

Supplementary Material 2

## Data Availability

All data generated or analysed during this study are included in this published article and its supplementary information files.
