# Supplementary material for: Trends over the past 15 years in long-term care in Switzerland: a comparison with Germany, Italy, Norway, and the United Kingdom
Source: BMC Geriatr. 2024 Jul 23;24:627. doi: 10.1186/s12877-024-05195-8 (PMC11265318; doi:10.1186/s12877-024-05195-8)
Supplement: Supplementary file 1 — Supplementary Material 1 [file 12877_2024_5195_MOESM1_ESM.docx]

**Trends over the past 15 years in long-term care in Switzerland: a comparison with Germany, Italy, Norway, and the United Kingdom**

***Appendix A***

| Table S1. Population statistics and health expenditure | | | | | |
| --- | --- | --- | --- | --- | --- |
| **Year** | **Switzerland** | **Germany** | **Italy** | **Norway** | **United Kingdom** |
| *Population* | | | | | |
| **Total population** | | | | | |
| 2005 | 7'437'115 | 82'469'422 | 57'969'484 | 4'623'291 | 60'401'206 |
| 2010 | 7'824'909 | 81'776'930 | 59'277'417 | 4'889'252 | 62'766'365 |
| 2015 | 8'282'396 | 81'686'611 | 60'730'582 | 5'188'607 | 65'116'219 |
| 2019 | 8'575'280 | 83'092'962 | 59'729'081 | 5'347'896 | 66'836'327 |
| **Population 64 and under, n (% of total population)** | | | | | |
| 2005 | 6'263'273 (84.2) | 66'908'824 (81.1) | 46'572'819 (80.3) | 3'938'349 (85.2) | 50'715'497  (84.0) |
| 2010 | 6'503'175 (83.1) | 64'969'522 (79.4) | 47'168'915 (79.6) | 4'161'373 (85.1) | 52'366'139  (83.4) |
| 2015 | 6'791'786 (82.0) | 64'353'863 (78.8) | 47'401'041 (78.1) | 4'342'707 (83.7) | 53'412'093  (82.0) |
| 2019 | 6'959'869 (81.2) | 65'175'633 (78.4) | 45'984'144 (77.0) | 4'424'172 (82.7) | 54'465'794  (81.5) |
| **Population 65 and over, n (% of total population)** | | | | | |
| 2005 | 1'173'842 (15.8) | 15'560'598 (18.9) | 11'396'665 (19.7) | 684'943 (14.8) | 9'685'709  (16.0) |
| 2010 | 1'321'734 (16.9) | 16'807'408 (20.6) | 12'108'503 (20.4) | 727'879 (14.9) | 10'400'226  (16.6) |
| 2015 | 1'490'610 (18.0) | 17'332'748 (21.2) | 13'329'540 (21.9) | 845'900 (16.3) | 11'704'126  (18.0) |
| 2019 | 1'615'411 (18.8) | 17'917'329 (21.6) | 13'744'938 (23.0) | 923'724 (17.3) | 12'370'532  (18.5) |
| *Expenditure* | | | | | |
| **GDP, per capita (current prices, current ppp, USD)** | | | | | |
| 2005 | 41'245 | 32'237 | 30'028 | 47'801 | 32'741 |
| 2010 | 54'352 | 39'677 | 34'840 | 57'923 | 36'585 |
| 2015 | 65'265 | 47'610 | 37'206 | 60'353 | 42'602 |
| 2019 | 71'010 | 55'651 | 44'377 | 66'798 | 48'702 |
| **Total health expenditure, as % of GDP** | | | | | |
| 2005 | 10.0 | 10.3 | 8.3 | 8.3 | 8.4 |
| 2010 | 9.9 | 11.1 | 8.9 | 8.9 | 10.0 |
| 2015 | 11.0 | 11.2 | 8.9 | 10.1 | 9.8 |
| 2019 | 11.3 | 11.7 | 8.7 | 10.5 | 9.9 |
| **Total health expenditure, per capita (current prices, current ppp, USD)** | | | | | |
| 2005 | 4'106.1 | 3'430.0 | 2'504.1 | 3'740.9 | 2'731.4 |
| 2010 | 5'089.2 | 4'423.1 | 3'104.5 | 4'777.1 | 3'441.7 |
| 2015 | 6'465.9 | 5'296.0 | 3'088.9 | 5'726.9 | 3'805.8 |
| 2019 | 6'942.4 | 6'407.9 | 3'565.3 | 6'476.4 | 4'385.5 |

| Table S2. Need for long-term care | | | | | |
| --- | --- | --- | --- | --- | --- |
| **Year** | **Switzerland** | **Germany** | **Italy** | **Norway** | **United Kingdom** |
| *Past need of long-term care* | | | | | |
| **Life expectancy at birth** | | | | | |
| 2000 | 80.0 | 78.3 | 79.9 | 78.8 | 77.9 |
| 2005 | 81.5 | 79.4 | 80.9 | 80.3 | 79.2 |
| 2010 | 82.7 | 80.5 | 82.2 | 81.2 | 80.6 |
| 2015 | 83.0 | 80.7 | 82.7 | 82.4 | 81.0 |
| 2019 | 84.0 | 81.3 | 83.6 | 83.0 | 81.4 |
| **Healthy life expectancy (HALE) at birth** | | | | | |
| 2000 | 69.3 | 68.5 | 69.0 | 68.4 | 67.6 |
| 2010 | 71.4 | 70.0 | 71.1 | 70.2 | 69.4 |
| 2015 | 71.7 | 70.1 | 71.6 | 71.2 | 69.7 |
| 2019 | 72.5 | 70.9 | 71.9 | 71.4 | 70.1 |
| **Proportion of 65-year-olds and over receiving long-term care in institutions** | | | | | |
| 2005 | 77'290 (6.6) | 585'347 (3.8) | - | 38'857 (5.7) | 281'000 (3.5) *(E)* |
| 2010 | 81'717 (6.2) | 645'941 (3.8) | - | 40'264 (5.5) | 246'000 (2.9) *(E)* |
| 2015 | 85'928 (5.8) | 702'466 (4.1) | - | 38'500 (4.6) | 228'185 (2.3) *(E)* |
| 2019 | 87'562 (5.4) | 742'366 (4.1) | - | 37'522 (4.1) | 218'485 (2.1) *(E)* |
| **Proportion of 65-year-olds and over receiving long-term care at home** | | | | | |
| 2005 | 145'046 (12.4) | 1'059'743 (6.8) | 334'238 (2.9) | 80'728 (11.8) | 1'020'000 (12.7) *(E)* |
| 2010 | 184'227 (13.9) | 1'287'498 (7.7) | 501'701 (4.1) | 90'025 (12.4) | 873'000 (10.2) *(E)* |
| 2015 | 230'653 (15.5) | 1'585'912 (9.1) | 732'825 (5.5) | 95'879 (11.3) | 359'275 (3.7) *(E)* |
| 2019 | 280'035 (17.3) | 2'547'404 (14.2) | 858'896 (6.2) | 100'807 (10.9) | 329'790 (3.2) *(E)* |
| **Proportion of 64-year-olds and under receiving long-term care in institutions** | | | | | |
| 2005 | 3'173 (0.05) | 96'212 (0.14) | - | 1'862 (0.05) | 65'000 (0.21) *(E)** |
| 2010 | 4'224 (0.06) | 107'301 (0.17) | - | 4'135 (0.10) | 54'000 (0.16) *(E)** |
| 2015 | 4'520 (0.07) | 105'483 (0.16) | - | 4'350 (0.10) | 47'450 (0.14) *(E)** |
| 2019 | 4'884 (0.07) | 170'868 (0.26) | - | 4'367 (0.10) | 45'560 (0.13) *(E)** |
| **Proportion of 64-year-olds and under receiving long-term care at home** | | | | | |
| 2005 | 50'171 (0.8) | 338'994 (0.5) | 62'519 (0.1) | 35'882 (0.9) | 472'000 (1.5) *(E)** |
| 2010 | 61'409 (0.9) | 389'755 (0.6) | 95'450 (0.2) | 56'397 (1.4) | 467'000 (1.4) *(E)** |
| 2015 | 79'048 (1.2) | 449'323 (0.7) | 155'179 (0.3) | 65'131 (1.5) | 237'505 (0.7) *(E)** |
| 2019 | 114'408 (1.6) | 790'988 (1.2) | 188'327 (0.4) | 72'909 (1.7) | 244'295 (0.6) *(E)** |
| *Future need of long-term care* | | | | | |
| **Population projections, in millions** | | | | | |
| 2030 | 9.4 | 83.4 | 59.9 | 5.8 | 69.2 |
| 2040 | 10.0 | 83.2 | 59.3 | 6.1 | 70.4 |
| 2050 | 10.4 | 82.6 | 58.0 | 6.4 | - |
| 2060 | - | 81.8 | 55.9 | 6.6 | - |
| **Number of people to receive care in an institution** | | | | | |
| 2030 | 93'786 | 958'000 | 704'000 | 60'000 | 305'000 |
| 2040 | 145'760** | 1'038'000 | 783'000 | 80'000 | 339'000 |
| 2050 | - | 1'234'000 | 891'000 | 96'000 | 360'000 |
| 2060 | - | 1'264'000 | 934'000 | 112'000 | 379'000 |
| **Number of people to receive care at home** | | | | | |
| 2030 | 400'000 | 814'000 | 832'000 | 254'000 | 1'264'000 |
| 2040 | - | 895'000 | 966'000 | 299'000 | 1'422'000 |
| 2050 | - | 995'000 | 1'133'000 | 337'000 | 1'534'000 |
| 2060 | - | 979'000 | 1'188'000 | 368'000 | 1'605'000 |
| *Indicator is for ages 18-64 rather than 0-64  **Indicator is for population over 65 rather than whole population | | | | | |

| Table S3. Health-related long-term care financing | | | | | |
| --- | --- | --- | --- | --- | --- |
| **Year** | **Switzerland** | **Germany** | **Italy** | **Norway** | **United Kingdom** |
| **Total expenditure on (health-related) long-term care** | | | | | |
| *As share of GDP* | | | | | |
| 2005 | 2.0 | 1.5 | - | 2.2 | - |
| 2010 | 2.0 | 1.7 | - | 2.5 | - |
| 2015 | 2.2 | 1.8 | 0.9 | 2.8 | 1.7 |
| 2019 | 2.3 | 2.2 | 0.9 | 3.1 | 1.8 |
| *As share of health expenditure* | | | | | |
| 2005 | 19.8 | 14.9 | - | 25.9 | - |
| 2010 | 20.1 | 15.2 | - | 28.5 | - |
| 2015 | 20.3 | 16.4 | 10.4 | 27.8 | 17.8 |
| 2019 | 20.4 | 19.2 | 10.4 | 29.7 | 17.9 |
| *Per capita (current prices, current ppp, USD)* | | | | | |
| 2005 | 811.3 | 511.9 | - | 967.5 | - |
| 2010 | 1024.2 | 671.6 | - | 1361.4 | - |
| 2015 | 1315.3 | 869.4 | 320.0 | 1592.2 | 679.0 |
| 2019 | 1418.3 | 1231.2 | 369.1 | 1922.0 | 801.6 |
| **Public expenditure on (health-related) long-term care (government and compulsory schemes)** | | | | | |
| *As share of GDP* | | | | | |
| 2005 | 1.2 | 1.0 | - | 1.9 | - |
| 2010 | 1.3 | 1.1 | - | 2.3 | - |
| 2015 | 1.5 | 1.3 | 0.7 | 2.6 | 1.2 |
| 2019 | 1.5 | 1.6 | 0.7 | 2.9 | 1.2 |
| *As share of health expenditure* | | | | | |
| 2005 | 12.1 | 9.7 | - | 23.0 | - |
| 2010 | 12.9 | 10.1 | - | 25.7 | - |
| 2015 | 13.5 | 11.2 | 7.8 | 26.2 | 12.1 |
| 2019 | 13.3 | 13.5 | 7.7 | 27.2 | 11.7 |
| *Per capita (current prices, current ppp, USD)* | | | | | |
| 2005 | 496.4 | 334.3 | - | 859.3 | - |
| 2010 | 659.0 | 448.1 | - | 1228.1 | - |
| 2015 | 875.3 | 595.3 | 240.1 | 1455.7 | 461.1 |
| 2019 | 926.3 | 864.9 | 274.3 | 1774.7 | 519.2 |
| **Household out-of-pocket payments on (health-related) long-term care** | | | | | |
| *As share of GDP* | | | | | |
| 2005 | 0.8 | 0.4 | - | 0.2 | - |
| 2010 | 0.7 | 0.5 | - | 0.2 | - |
| 2015 | 0.7 | 0.5 | 0.2 | 0.2 | 0.5 |
| 2019 | 0.8 | 0.6 | 0.2 | 0.2 | 0.5 |
| *As share of health expenditure* | | | | | |
| 2005 | 7.5 | 4.1 | - | 2.9 | - |
| 2010 | 7.0 | 4.1 | - | 2.8 | - |
| 2015 | 6.7 | 4.3 | 2.6 | 2.4 | 5.0 |
| 2019 | 6.9 | 5.0 | 2.6 | 2.3 | 5.4 |
| *Per capita (current prices, current ppp, USD)* | | | | | |
| 2005 | 309.9 | 142.1 | - | 108.2 | - |
| 2010 | 357.9 | 181.4 | - | 133.3 | - |
| 2015 | 431.1 | 230.2 | 78.8 | 136.5 | 191.2 |
| 2019 | 481.8 | 320.0 | 93.0 | 147.3 | 238.0 |
| **Projected public expenditure on (health-related) long-term care, as % of GDP** | | | | | |
| 2030 | 2.0 | 1.7 | 1.9 | 4.9 | 1.3 |
| 2040 | 2.6 | 1.8 | 2.2 | 5.9 | 1.4 |
| 2050 | 3.2 | 1.9 | 2.6 | 6.6 | 1.5 |
| 2060 | - | 1.9 | 2.8 | 7.4 | 1.5 |

| Table S4. Long-term care service delivery | | | | | |
| --- | --- | --- | --- | --- | --- |
| **Year** | **Switzerland** | **Germany** | **Italy** | **Norway** | **United Kingdom** |
| **Long-term care recipients in institutions (other than hospitals), n (% of total population)** | | | | | |
| 2005 | 80'463 (1.08) | 681'559 (0.83) | 292'141 (0.50) | 40'719 (0.88) | - |
| 2010 | 85'941 (1.10) | 753'242 (0.92) | 386'266 (0.65) | 44'399 (0.91) | - |
| 2015 | 90'448 (1.09) | 807'949 (0.98) | 447'193 (0.74) | 42'850 (0.82) | - |
| 2019 | 92'446 (1.08) | 913'234 (1.10) | 479'786 (0.80) | 41'889 (0.78) | 490'326 (0.73) *(2020)* |
| **Beds in institutional LTC facilities, n (per 1000 population aged 65 and over)** | | | | | |
| 2005 | 86'798 (73.9) | 757'186 (49.3) | 169'827 (15) | 41'027 (60.5) | 540'821 (56.2) |
| 2010 | 91'781 (70.1) | 875'549 (52.8) *(2011)* | 212'875 (17.6) | 41'297 (57.1) | 524'609 (51.1) |
| 2015 | 96'563 (65.9) | 928'939 (54.4) | 244'395 (18.5) | 40'708 (48.8) | 548'397 (47.2) |
| 2019 | 100'356 (63.6) | 969'553 (54.2) | 257'410 (18.8) | 39'963 (43.5) | 525'704 (42.5) |
| **Long-term care recipients at home, n (% of total population)** | | | | | |
| 2005 | 195'217 (2.6) | 1'398'737 (1.7) | 396'757 (0.7) | 116'610 (2.5) | 1'708'000 (2.8) *(E)* |
| 2010 | 245'636 (3.1) | 1'677'253 (2.1) | 597'151 (1.0) | 146'422 (3.0) | - |
| 2015 | 309'098 (3.7) | 2'035'235 (2.5) | 888'004 (1.5) | 161'011 (3.1) | - |
| 2019 | 394'444 (4.6) | 3'338'392 (4.0) | 1'047'223 (1.8) | 173'716 (3.3) | 957'831 (1.4) |

| Table S5. Long-term care workforce | | | | | |
| --- | --- | --- | --- | --- | --- |
| **Year** | **Switzerland** | **Germany** | **Italy** | **Norway** | **United Kingdom** |
| *Long-term care formal sector workforce* | | | | | |
| **Total nurses and personal carers, n (% of population aged 65 and over)** | | | | | |
| 2005 | 82'421 (6.9) *(2006)* | 585'948 (3.8) | - | - | - |
| 2010 | 99'403 (7.6) | 745'932 (4.5) *(2011)* | - | 95'663 (13.2) | - |
| 2015 | 116'775 (8.0) | 864'082 (5.1) | - | 106'941 (12.8) | - |
| 2019 | 131'141 (8.3) | 974'138 (5.4) | - | 113'766 (12.4) | 1'280'000 (12.2) *(2020) (E)* |
| **Total nurses and personal carers working in long-term care institutions, n (% of population aged 65 and over)** | | | | | |
| 2005 | 58'035 (4.9) *(2006)* | 394'369 (2.6) | - | - | - |
| 2010 | 66'907 (5.1) | 486'374 (2.9) *(2011)* | 155'104 (1.3) | - | - |
| 2015 | 76'233 (5.2) | 547'923 (3.2) | 145'457 (1.1) | - | - |
| 2019 | 82'921 (5.3) | 566'536 (3.3) | 158'601 (1.2) | - | 696'340 (5.6) |
| **Total nurses and personal carers working in long-term home care, n (% of population aged 65 and over)** | | | | | |
| 2005 | 24'000 (2.0) | 191'579 (1.2) | - | - | - |
| 2010 | 32'496 (2.5) | 259'558 (1.6) *(2011)* | - | - | - |
| 2015 | 40'542 (2.8) | 316'159 (1.9) | - | - | - |
| 2019 | 48'220 (3.1) | 380'994 (2.1) | - | - | 822'961 (6.7) |
| *Long-term care informal sector workforce* | | | | | |
| **Proportion of informal carers among population aged 50 and over** | | | | | |
| 2007 | 10.8 | 11 | 16.2 | - | 15.2 |
| 2010 | 14.8 | 15.7 | 19.7 | - | 18.2 *(2009)* |
| 2015 | 11 | 15 | 11 | - | 17 *(E)* |
| 2019 | 15 | 18 | 11 | 16* | 18 *(2017) (E)* |
| * Indicator is percent of unpaid care work among persons 16 years and over rather than 50 and over | | | | | |
