# Supplementary material for: Trends over the past 15 years in long-term care in Switzerland: a comparison with Germany, Italy, Norway, and the United Kingdom
Source: BMC Geriatr. 2024 Jul 23;24:627. doi: 10.1186/s12877-024-05195-8 (PMC11265318; doi:10.1186/s12877-024-05195-8)
Supplement: Supplementary file 2 — Supplementary Material 2 [file 12877_2024_5195_MOESM2_ESM.docx]

**Trends over the past 15 years in long-term care in Switzerland: a comparison with Germany, Italy, Norway, and the United Kingdom**

***Appendix B***

| **Table 1: Sources** | | | | | |
| --- | --- | --- | --- | --- | --- |
| **Country** | **Switzerland** | **Germany** | **Italy** | **Norway** | **United Kingdom** |
| **Source: Eligibility criteria for long-term care** | Trein, P. (2018). ESPN Thematic Report on Challenges in long-term care: Switzerland, European Commission, The European Social Policy Network. | Blümel, M., et al. (2020). Germany: Health System Review. Health Systems in Transition, The European Observatory on Health Systems and Policies. **22**.  Gerlinger, T. (2018). ESPN Thematic Report on Challenges in Long-Term Care: Germany, European Commission, The European Social Policy Network. | Jessoula, M., et al. (2018). ESPN Thematic Report on Challenges in long-term care: Italy, European Commission, The European Social Policy Network.    Ferre, F., et al. (2014). "Italy: Health System Review." Health Syst Transit **16**(4): 1-168. | Saunes, I. S., et al. (2020). Norway: Health System Review. Health Systems in Transition, The European Observatory on Health Systems and Policies.    Sandvin, J. T. and T. Bliksvaer (2019). Task 2017-18 Disability assessment – country report, The Academic Network of European Disability Experts. | gov.uk (2016). "Guidance: Care Act factsheets." from https://www.gov.uk/government/publications/care-act-2014-part-1-factsheets/care-act-factsheets#factsheet-2-who-is-entitled-to-public-care-and-support. |
| **Source: Publicly available long-term care services** | De Pietro, C., et al. (2015). "Switzerland: Health System Review." Health Syst Transit **17**(4): 1-288, xix. | Blümel, M., et al. (2020). Germany: Health System Review. Health Systems in Transition, The European Observatory on Health Systems and Policies. **22**.  Gerlinger, T. (2018). ESPN Thematic Report on Challenges in Long-Term Care: Germany, European Commission, The European Social Policy Network. | Jessoula, M., et al. (2018). ESPN Thematic Report on Challenges in long-term care: Italy, European Commission, The European Social Policy Network.    Ferre, F., et al. (2014). "Italy: Health System Review." Health Syst Transit **16**(4): 1-168. | Saunes, I. S., et al. (2020). Norway: Health System Review. Health Systems in Transition, The European Observatory on Health Systems and Policies.  Grødem, A. S. (2018). ESPN Thematic Report on Challenges in long-term care: Norway, European Commission, The European Social Policy Network. | Glendinning, C. (2018). ESPN Thematic Report on Challenges in long-term care: United Kingdom, European Commission, The European Social Policy Network.    Anderson, M., et al. (2022). United Kingdom: Health System Review, The European Observatory on Health Systems and Policies. **24**. |

| **Indicator** | **Source** | **Original Source/Years** | **Definition** | **Missing Data** |
| --- | --- | --- | --- | --- |
| **Population statistics, expenditure and insurance coverage** | | | | |
| Total population | The World Bank Data Bank : World Development Indicators. | **Switzerland (2005-2019):** Eurostat  **Germany (2005-2019):** Eurostat, Federal Statistical Office  **Italy (2005-2019):** Eurostat  **Norway** **(2005-2019):** Eurostat, Statistics Norway  **United Kingdom** **(2005-2019):**  Eurostat, Office for National Statistics | Total population is based on the de facto definition of population, which counts all residents regardless of legal status or citizenship. The values shown are midyear estimates. | N/A |
| Population under 64 | The World Bank Data Bank : World Development Indicators. | World Bank staff estimates using the World Bank's total population and age/sex distributions of the United Nations Population Division's World Population Prospects: 2019 Revision. | Total population between the ages 0 to 14. Population is based on the de facto definition of population, which counts all residents regardless of legal status or citizenship.  Total population between the ages 15 to 64. Population is based on the de facto definition of population, which counts all residents regardless of legal status or citizenship.  Population 64 and under was calculated via the addition of the total population between 0 to 14 and 15 to 64. Proportions were calculated via the division of the population 64 and under by the total population. | N/A |
| Population over 65 | The World Bank Data Bank : World Development Indicators. | World Bank staff estimates using the World Bank's total population and age/sex distributions of the United Nations Population Division's World Population Prospects: 2019 Revision. | Total population 65 years of age or older. Population is based on the de facto definition of population, which counts all residents regardless of legal status or citizenship. | N/A |
| GDP, per capita (current prices, current ppp, USD) | OECD Statistics : National Accounts : Annual National Accounts : Main Aggregates. | National Account database (OECD) | Gross domestic product (GDP) is the standard measure of the value added created through the production of goods and services in a country during a certain period. As such, it also measures the income earned from that production, or the total amount spent on final goods and services (less imports). While GDP is the single most important indicator to capture economic activity, it falls short of providing a suitable measure of people's material well-being for which alternative indicators may be more appropriate. This indicator is based on nominal GDP (also called GDP at current prices or GDP in value) and is available in US dollars per capita (current PPPs). All OECD countries compile their data according to the 2008 System of National Accounts (SNA). This indicator is less suited for comparisons over time, as developments are not only caused by real growth, but also by changes in prices and PPPs. | Data is national, but for Norway's GDP, the coverage is Norway mainland.  For countries where GDP is not reported for the same reference period as data on educational finance, GDP is estimated as: wt-1 (GDPt - 1) + wt (GDPt), where wt and wt-1 are the weights for the respective portions of the two reference periods for GDP which fall within the educational financial year. Adjustments were made for the United Kingdom. |
| Total health expenditure | OECD Statistics : Health : Health expenditure and financing. | Joint OECD, EUROSTAT and WHO Health Accounts SHA Questionnaires (JHAQ) | All financing schemes: Government payments, compulsory schemes, voluntary schemes and household out-of-pocket payments. Current expenditure on health in all functions: inpatient curative and rehabilitative care, outpatient curative and rehabilitative care, long-term care (health), ancillary services (non-specified by function), medical goods (non-specified by function), preventive care, governance and health system and financing administration, other health care services unknown. All providers: hospitals, residential long-term care facilities, providers of ambulatory care, providers of ancillary services, retailers and other providers of medical goods, providers of preventive care, providers of health care system administration and financing, rest of the economy, rest of the world, providers unknown.  The approach taken in SHA 1.0 was to sum up the two aggregates of “current expenditure on health” and “gross capital formation” to arrive at “total health expenditure”. However, the use of the aggregate “total health expenditure” tended to be misunderstood. In effect, while current health expenditure refers to final consumption, which is the demand for health care goods and services by households, government and non-profit institutions, gross capital formation refers to the demand for capital goods by health providers. Thus, it could be argued that the two aggregates cannot be directly summed up as they refer to different timings of consumption, since capital formation enables future provision. For this reason, it is recommended to keep the two aggregates “current expenditure on health care” and “gross capital formation in health care” separate under SHA 2011, and to discourage the use of the aggregate “total health expenditure”, at least with respect to how it was used in SHA 1.0. | N/A |
| **Need for long-term care** | | | | |
| Life expectancy at birth | OECD Statistics : Health: Health Status. | **Switzerland, Germany, Italy, Norway (2000-2019):** Eurostat database  **United Kingdom (2000-2019):** Office of National Statistics | Life expectancy at birth and at ages 40, 60, 65 and 80 years old is the average number of years that a person at that age can be expected to live, assuming that age-specific mortality levels remain constant. | N/A |
| Healthy life expectancy (HALE) at birth | World Health Organization : The Global Health Observatory : Healthy life expectancy (HALE) at birth (years). | **Switzerland, Germany, Italy, Norway, United Kingdom (2000-2019):** These estimates draw on new data and on the results of the GBD 2010 study | Average number of years that a person can expect to live in "full health" by taking into account years lived in less than full health due to disease and/or injury.  The equivalent lost healthy year fractions required for the HALE calculation are estimated as the all-cause years lost due to disability (YLD) rate per capita, adjusted for independent comorbidity, by age, sex and country. Sullivan's method uses the equivalent lost healthy year fraction (adjusted for comorbidity) at each age in the current population (for a given year) to divide the hypothetical years of life lived by a period life table cohort at different ages into years of equivalent full health and equivalent lost healthy years . Predominant type of statistics: Predicted  Because these estimates draw on new data and on the results of the GBD 2010 study, and there have been substantial revisions to methods for many causes, and to the methods for dealing with comorbidity, these HALE estimates for the years 2000-2012 are not directly comparable with previous WHO estimates of HALE for earlier years. | **Switzerland, Germany, Italy, Norway, United Kingdom (2005):** Data unavailable |
| Proportion of 65-year-olds and over receiving long-term care in institutions | OECD Statistics :  Health : Long-term care resources and utilization : Long-term care recipients. | **Switzerland (2005-2019):** Federal Statistical Office  **Germany (2005-2019):** Federal Ministry of Health, Association of Private Health Insurance, Federal Statistical Office  **Norway (2005):** Statistics Norway, Official Statistics of Norway: Nursing and Care Statistics (Annual survey of municipal services (KOSTRA))  **Norway (2010-2015):** IPLOS register. National statistics produced by Statistics Norway  **Norway (2019):** The Municipal Patient and User Registry, Norwegian Directorate of Health | [Please refer to indicator 'Long-term care recipients in institutions (other than hospitals)' for definition and classification of recipients of long-term care in institutions. Same definitions and classifications apply here, but applied for the population of 65-year-olds and over] | **Italy (2005-2019):** Data available only for the total population (all ages)  **Norway (2005):** Before 2007, age-breakdown is sometimes different from the OECD guideline, and data are available for the recipients of all ages and those below 67, aged 67 and over and aged 80 and over.  **Norway (2005-2019):** Recipients of short term care in institutions are included in the figures for recipients of long term care in  institutions and not included among the recipients of long term care at home.  **UK (2005):**  Data for long-term recipients of 65 years and older in institutions other than hospitals was unavailable on OECD Statistics for the UK. UK data for 2005 to match this indicator was sourced from *The Information Centre for Knowledge for Care, (2005-06). Data on Community Care Statistics 2005-06: Referrals, Assessments and Packages of Care, England - National Summary.*  <https://view.officeapps.live.com/op/view.aspx?src=https%3A%2F%2Ffiles.digital.nhs.uk%2Fpublicationimport%2Fpub01xxx%2Fpub01491%2Fcomm-care-ref-eng-05-06-asse-pack-sum-tab.xls&wdOrigin=BROWSELINK>  Data was taken from Table 3 and includes Residential Care (independent sector residential care, LA staffed residential care, nursing care) from years 2005-2006 and population 65 and over.  **UK (2010):**  Data for long-term recipients of 65 years and older in institutions other than hospitals was unavailable on OECD Statistics for the UK. UK data for 2010 to match this indicator was sourced from *The Information Centre for Health and Social Care (2012). Community Care Statistics, Social Services Activity, England 2010-2011, Final Release: National Tables.* <https://view.officeapps.live.com/op/view.aspx?src=https%3A%2F%2Ffiles.digital.nhs.uk%2Fpublicationimport%2Fpub05xxx%2Fpub05264%2Fcomm-care-soci-serv-act-eng-10-11-fin-tab.xls&wdOrigin=BROWSELINK>  Data was taken from the Table for Packages of Care and includes Residential care and nursing care from years 2010-2011 and population 65 and over.  **UK (2015):**  Data for long-term recipients of 65 years and older in institutions other than hospitals was unavailable on OECD Statistics for the UK. UK data for 2015 to match this indicator was sourced from *NHS Digital (2016). Community Care Statistics, Social Services Activity, England 2015-2016, Final Release: National Tables.*  <https://view.officeapps.live.com/op/view.aspx?src=https%3A%2F%2Ffiles.digital.nhs.uk%2Fpublicationimport%2Fpub21xxx%2Fpub21934%2Fcomm-care-stat-act-eng-2015-16-nat-eng.xlsx&wdOrigin=BROWSELINK>  Data was taken from the Table LTS001a and includes Residential care and nursing care from years 2015-2016 and population 65 and over.  **UK (2019):**  Data for long-term recipients of 65 years and older in institutions other than hospitals was unavailable on OECD Statistics for the UK. UK data for 2015 to match this indicator was sourced from *NHS Digital (2020). Adult Social Care Activity and Finance Report, England 2019-20: Reference Data Tables.*  <https://view.officeapps.live.com/op/view.aspx?src=https%3A%2F%2Ffiles.digital.nhs.uk%2FBF%2F7AEF16%2FASCFR%2520and%2520SALT%2520Reference%2520Tables%25202019-20.xlsx&wdOrigin=BROWSELINK>  Data was taken from the Table 34 and includes Residential care and nursing care from years 2019-2020 and population 65 and over. |
| Proportion of 65-year-olds and over receiving long-term care at home | OECD Statistics :  Health : Long-term care resources and utilization : Long-term care recipients. | **Switzerland (2005):** Federal Office of Social Insurance  **Switzerland (2010-2019):** Federal Statistical Office  **Germany (2005-2019):** Federal Ministry of Health, Association of Private Health Insurance, Federal Statistical Office  **Italy (2005-2019):** Ministry of Health - Health Information System  **Norway (2005):** Statistics Norway, Official Statistics of Norway: Nursing and Care Statistics (Annual survey of municipal services (KOSTRA))  **Norway (2010-2019):** IPLOS register. National statistics produced by Statistics Norway | [Please refer to indicator 'Long-term care recipients at home' for definition and classification of recipients of long-term care in institutions. Same definitions and classifications apply here, but applied for the population of 65-year-olds and over] | **Italy (2005-2019):** Data available only for the total population (all ages)  **Norway (2005):** Before 2007, age-breakdown is sometimes different from the OECD guideline, and data are available for the recipients of all ages and those below 67, aged 67 and over and aged 80 and over.  **Norway (2005-2019):** Recipients of short term care in institutions are included in the figures for recipients of long term care in  institutions and not included among the recipients of long term care at home.  **UK (2005):**  Data for long-term recipients of 65 years and older at home other than hospitals was unavailable on OECD Statistics for the UK. UK data from 2005 to match this indicator was sourced from *The Information Centre for Knowledge for Care, (2005-06). Data on Community Care Statistics 2005-06: Referrals, Assessments and Packages of Care, England - National Summary.*  <https://view.officeapps.live.com/op/view.aspx?src=https%3A%2F%2Ffiles.digital.nhs.uk%2Fpublicationimport%2Fpub01xxx%2Fpub01491%2Fcomm-care-ref-eng-05-06-asse-pack-sum-tab.xls&wdOrigin=BROWSELINK>  Data was taken from Table 3 and includes Community-based services (day care, meals, home care, overnight respite, short-term residential, direct payments, professional support, transport, equipment and adaptations, other) from years 2005-2006 and population 65 and over.  **UK (2010):**  Data for long-term recipients of 65 years and older in institutions other than hospitals was unavailable on OECD Statistics for the UK. UK data from 2010 to match this indicator was sourced from *The Information Centre for Health and Social Care (2012). Community Care Statistics, Social Services Activity, England 2010-2011, Final Release: National Tables.*  <https://view.officeapps.live.com/op/view.aspx?src=https%3A%2F%2Ffiles.digital.nhs.uk%2Fpublicationimport%2Fpub05xxx%2Fpub05264%2Fcomm-care-soci-serv-act-eng-10-11-fin-tab.xls&wdOrigin=BROWSELINK>  Data was taken from the Table for Packages of Care and includes Community-based services (existing/new direct payments and personal budgets, direct payments, home care, day care, meals, short term residential, professional support, equipment and adaptations, other) from years 2010-2011 and population 65 and over.  **UK (2015):**  Data for long-term recipients of 65 years and older at home other than hospitals was unavailable on OECD Statistics for the UK. UK data from 2015 to match this indicator was sourced from *NHS Digital (2016). Community Care Statistics, Social Services Activity, England 2015-2016, Final Release: National Tables.*  <https://view.officeapps.live.com/op/view.aspx?src=https%3A%2F%2Ffiles.digital.nhs.uk%2Fpublicationimport%2Fpub21xxx%2Fpub21934%2Fcomm-care-stat-act-eng-2015-16-nat-eng.xlsx&wdOrigin=BROWSELINK>  Data was taken from the Table LTS001a and includes Community Direct Payment Only, Community Part Direct Payment, Community CASSR Managed Personal Budget, Community CASSR Commissioned Support Only, from years 2015-2016 and population 65 and over.  **UK (2019):**  Data for long-term recipients of 65 years and older at home other than hospitals was unavailable on OECD Statistics for the UK. UK data from 2019 to match this indicator was sourced from *NHS Digital (2020). Adult Social Care Activity and Finance Report, England 2019-20: Reference Data Tables.*  <https://view.officeapps.live.com/op/view.aspx?src=https%3A%2F%2Ffiles.digital.nhs.uk%2FBF%2F7AEF16%2FASCFR%2520and%2520SALT%2520Reference%2520Tables%25202019-20.xlsx&wdOrigin=BROWSELINK>  Data was taken from the Table 34 and includes Community Direct Payment Only, Community Part Direct Payment, Community CASSR Managed Personal Budget, Community CASSR Commissioned Support Only, from years 2019-2020 and population 65 and over. |
| Proportion of 64-year-olds and under receiving long-term care in institutions | OECD Statistics : Health : Long-term care resources and utilization : Long-term care recipients. | **Switzerland (2005-2019):** Federal Statistical Office  **Germany (2005-2019):** Federal Ministry of Health, Association of Private Health Insurance, Federal Statistical Office  **Norway (2005):** Statistics Norway, Official Statistics of Norway: Nursing and Care Statistics (Annual survey of municipal services (KOSTRA))  **Norway (2010-2015):** IPLOS register. National statistics produced by Statistics Norway  **Norway (2019):** The Municipal Patient and User Registry, Norwegian Directorate of Health | [Please refer to indicator 'Long-term care recipients in institutions (other than hospitals)' for definition and classification of recipients of long-term care in institutions. Same definitions and classifications apply here, but applied for the population of 64-year-olds and under] | **Italy (2005-2019):** Data available only for the total population (all ages)  **Norway (2005):** Before 2007, age-breakdown is sometimes different from the OECD guideline, and data are available for the recipients of all ages and those below 67, aged 67 and over and aged 80 and over.  **Norway (2005-2019):** Recipients of short term care in institutions are included in the figures for recipients of long term care in institutions and not included among the recipients of long term care at home.  **UK (2005):**  Data for long-term recipients of 64 years and under in institutions other than hospitals was unavailable on OECD Statistics for the UK. Data for UK represents a different indicator, proportion of 18-64 years-olds receiving long-term care in institutions.  UK data for this indicator for 2005 was sourced from  *The Information Centre for Knowledge for Care (2005-06). Data on Community Care Statistics 2005-06: Referrals, Assessments and Packages of Care, England - National Summary.*  <https://view.officeapps.live.com/op/view.aspx?src=https%3A%2F%2Ffiles.digital.nhs.uk%2Fpublicationimport%2Fpub01xxx%2Fpub01491%2Fcomm-care-ref-eng-05-06-asse-pack-sum-tab.xls&wdOrigin=BROWSELINK>  Data was taken from Table 3 and includes Residential Care (independent sector residential care, LA staffed residential care, nursing care) from years 2005-2006 and population 18-64.  **UK (2010):**  Data for long-term recipients of 64 years and under in institutions other than hospitals was unavailable on OECD Statistics for the UK. Data for UK represents a different indicator, proportion of 18-64 years-olds receiving long-term care in institutions.  UK data for this indicator for 2010 was sourced from  *The Information Centre for Health and Social Care (2012). Community Care Statistics, Social Services Activity, England 2010-2011, Final Release: National Tables.* <https://view.officeapps.live.com/op/view.aspx?src=https%3A%2F%2Ffiles.digital.nhs.uk%2Fpublicationimport%2Fpub05xxx%2Fpub05264%2Fcomm-care-soci-serv-act-eng-10-11-fin-tab.xls&wdOrigin=BROWSELINK>  Data was taken from the Table for Packages of Care and includes Residential care and nursing care from years 2010-2011 and population 18-64.  **UK (2015):**  Data for long-term recipients of 64 years and under in institutions other than hospitals was unavailable on OECD Statistics for the UK. Data for UK represents a different indicator, proportion of 18-64 years-olds receiving long-term care in institutions.  UK data for this indicator for 2015 was sourced from *NHS Digital (2016). Community Care Statistics, Social Services Activity, England 2015-2016, Final Release: National Tables.*  <https://view.officeapps.live.com/op/view.aspx?src=https%3A%2F%2Ffiles.digital.nhs.uk%2Fpublicationimport%2Fpub21xxx%2Fpub21934%2Fcomm-care-stat-act-eng-2015-16-nat-eng.xlsx&wdOrigin=BROWSELINK>  Data was taken from the Table LTS001a and includes Residential care and nursing care from years 2015-2016 and population 18-64.  **UK (2019):**  Data for long-term recipients of 64 years and under in institutions other than hospitals was unavailable on OECD Statistics for the UK. Data for UK represents a different indicator, proportion of 18-64 years-olds receiving long-term care in institutions.  UK data for this indicator for 2019 was sourced from *NHS Digital (2020). Adult Social Care Activity and Finance Report, England 2019-20: Reference Data Tables.*  <https://view.officeapps.live.com/op/view.aspx?src=https%3A%2F%2Ffiles.digital.nhs.uk%2FBF%2F7AEF16%2FASCFR%2520and%2520SALT%2520Reference%2520Tables%25202019-20.xlsx&wdOrigin=BROWSELINK>  Data was taken from the Table 34 and includes Residential care and nursing care from years 2019-2020 and population 18-64. |
| Proportion of 64-year-olds and under receiving long-term care at home | OECD Statistics :  Health : Long-term care resources and utilization : Long-term care recipients. | **Switzerland (2005):** Federal Office of Social Insurance  **Switzerland (2010-2019):** Federal Statistical Office  **Germany (2005-2019):** Federal Ministry of Health, Association of Private Health Insurance, Federal Statistical Office  **Italy (2005-2019):** Ministry of Health - Health Information System  **Norway (2005):** Statistics Norway, Official Statistics of Norway: Nursing and Care Statistics (Annual survey of municipal services (KOSTRA))  **Norway (2010-2019):** IPLOS register. National statistics produced by Statistics Norway | [Please refer to indicator 'Long-term care recipients at home' for definition and classification of recipients of long-term care in institutions. Same definitions and classifications apply here, but applied for the population of 64-year-olds and under] | **Italy (2005-2019):** Data available only for the total population (all ages)  **Norway (2005):** Before 2007, age-breakdown is sometimes different from the OECD guideline, and data are available for the recipients of all ages and those below 67, aged 67 and over and aged 80 and over.  **UK (2005):**  Data for long-term recipients of 64 years and under at home was unavailable on OECD Statistics for the UK. Data for UK represents a different indicator, proportion of 18-64 years-olds receiving long-term care at home. UK data for this indicator for 2005 was sourced from *The Information Centre for Knowledge for Care, (2005-06). Data on Community Care Statistics 2005-06: Referrals, Assessments and Packages of Care, England - National Summary.*  <https://view.officeapps.live.com/op/view.aspx?src=https%3A%2F%2Ffiles.digital.nhs.uk%2Fpublicationimport%2Fpub01xxx%2Fpub01491%2Fcomm-care-ref-eng-05-06-asse-pack-sum-tab.xls&wdOrigin=BROWSELINK>  Data was taken from Table 3 and includes Community-based services (day care, meals, home care, overnight respite, short-term residential, direct payments, professional support, transport, equipment and adaptations, other) from years 2005-2006 and population 18-64.  **UK (2010):**  Data for long-term recipients of 64 years and under in institutions other than hospitals was unavailable on OECD Statistics for the UK. Data for UK represents a different indicator, proportion of 18-64 years-olds receiving long-term care in institutions. UK data for this indicator for 2010 was sourced from *The Information Centre for Health and Social Care (2012). Community Care Statistics, Social Services Activity, England 2010-2011, Final Release: National Tables.*  <https://view.officeapps.live.com/op/view.aspx?src=https%3A%2F%2Ffiles.digital.nhs.uk%2Fpublicationimport%2Fpub05xxx%2Fpub05264%2Fcomm-care-soci-serv-act-eng-10-11-fin-tab.xls&wdOrigin=BROWSELINK>  Data was taken from the Table for Packages of Care and includes Community-based services (existing/new direct payments and personal budgets, direct payments, home care, day care, meals, short term residential, professional support, equipment and adaptations, other) from years 2010-2011 and population 18-64.  **UK (2015):**  Data for long-term recipients of 64 years and under in institutions other than hospitals was unavailable on OECD Statistics for the UK. Data for UK represents a different indicator, proportion of 18-64 years-olds receiving long-term care in institutions. UK data for this indicator for 2015 was sourced from *NHS Digital (2016). Community Care Statistics, Social Services Activity, England 2015-2016, Final Release: National Tables.*  <https://view.officeapps.live.com/op/view.aspx?src=https%3A%2F%2Ffiles.digital.nhs.uk%2Fpublicationimport%2Fpub21xxx%2Fpub21934%2Fcomm-care-stat-act-eng-2015-16-nat-eng.xlsx&wdOrigin=BROWSELINK>  Data was taken from the Table LTS001a and includes Community Direct Payment Only, Community Part Direct Payment, Community CASSR Managed Personal Budget, Community CASSR Commissioned Support Only, from years 2015-2016 and population 18-64.  **UK (2019):**  Data for long-term recipients of 64 years and under in institutions other than hospitals was unavailable on OECD Statistics for the UK. Data for UK represents a different indicator, proportion of 18-64 years-olds receiving long-term care in institutions. UK data for this indicator for 2019 was sourced from *NHS Digital (2020). Adult Social Care Activity and Finance Report, England 2019-20: Reference Data Tables.*  <https://view.officeapps.live.com/op/view.aspx?src=https%3A%2F%2Ffiles.digital.nhs.uk%2FBF%2F7AEF16%2FASCFR%2520and%2520SALT%2520Reference%2520Tables%25202019-20.xlsx&wdOrigin=BROWSELINK>  Data was taken from the Table 34 and includes Community Direct Payment Only, Community Part Direct Payment, Community CASSR Managed Personal Budget, Community CASSR Commissioned Support Only, from years 2019-2020 and population 18-64. |
| Population projections, in millions | European Commission Directorate-General for Economic Financial Affairs. The 2021 ageing report : economic & budgetary projections for the EU Member States (2019-2070). Publications Office; 2021. | **Germany, Italy, Norway (2030-2060):** European Commission (DG ECFIN), Eurostat (EUROPOP2019), EPC (AWG) | Reference scenario for the projected population of each country in given years. | **Switzerland (2030-2050):** Data unavailable in 2021 Ageing Report of European Commission. Swiss data to match this indicator was sourced from the *Federal Statistical Office (2021). Population projections: National projections.*  <https://www.bfs.admin.ch/bfs/en/home/statistics/population/population-projections/national-projections.html>  **United Kingdom (2030-2040):** Data unavailable in 2021 Ageing Report of European Commission. UK data to match this indicator was sourced from the *Office for National Statistics National population projections.*  <https://www.ons.gov.uk/peoplepopulationandcommunity/populationandmigration/populationprojections/bulletins/nationalpopulationprojections/2020basedinterim> |
| Number of people to receive care in an institution | **Germany, Italy, Norway (2030-2060):**  European Commission Directorate-General for Economic Financial Affairs. The 2021 ageing report : economic & budgetary projections for the EU Member States (2019-2070). Publications Office; 2021.  **United Kingdom (2030-2060):** European Commission Directorate-General for Economic Financial Affairs. The 2015 ageing report : economic & budgetary projections for the EU Member States (2013-2060). 2015. | **Germany, Italy, Norway (2030-2060):**  European Commission (DG ECFIN), Eurostat (EUROPOP2019), EPC (AWG)  **United Kingdom (2030-2060):** Commission Services (DG ECFIN), Eurostat (EUROPOP2013), EPC (AWG) | Institutional care is provided in an institution where the recipient resides. | **Switzerland (2030):** Data unavailable in 2015 and 2021 Ageing Report of European Commission. Swiss data to match this indicator was sourced from Table 11 of *Swiss Health Observatory (2010). Statistische Grundlagen zur regionalen Pflegeheimplanung in der Schweiz: Methodik und kantonale Kennzahlen [Statistical bases for regional nursing home planning in Switzerland: Methodology and cantonal key figures].*  <https://www.obsan.admin.ch/sites/default/files/obsan_47_bericht.pdf>  **Switzerland (2040):** Data for the number of people to receive care in an institution in 2040 was unavailable for Switzerland. Swiss predictions for 2040 represent a different indicator, number of people 65 years old and above to receive care in an institution. Swiss data for this indicator was sourced from Table G5.1 of *Pellegrini, S., et al. (2022). Bedarf an Alters- und Langzeitpflege in der Schweiz [Demand for old-age and long-term care in Switzerland], Swiss Health Observatory.*  <https://www.obsan.admin.ch/sites/default/files/2022-05/Obsan_03_2022_BERICHT.pdf>  The projections are taken from the scenario for constant care duration. |
| Number of people to receive care at home | **Germany, Italy, Norway (2030-2060):**  European Commission Directorate-General for Economic Financial Affairs. The 2021 ageing report : economic & budgetary projections for the EU Member States (2019-2070). Publications Office; 2021.  **United Kingdom (2030-2060):** European Commission Directorate-General for Economic Financial Affairs. The 2015 ageing report : economic & budgetary projections for the EU Member States (2013-2060). 2015. | **Germany, Italy, Norway (2030-2060):**  European Commission (DG ECFIN), Eurostat (EUROPOP2019), EPC (AWG)  **United Kingdom (2030-2060):** Commission Services (DG ECFIN), Eurostat (EUROPOP2013), EPC (AWG) | Home care is provided in the private home of the care recipient. | **Switzerland (2030):** Data unavailable in 2015 and 2021 Ageing Report of European Commission. Swiss data to match this indicator was sourced from *Höpflinger, F., et al. (2011). Pflegebedürftigkeit und Langzeitpflege im Alter: Aktualisierte Szenarien für die Schweiz [Long-term care needs and long-term care in old age: Updated scenarios for Switzerland], Hans Huber.*  <https://www.obsan.admin.ch/sites/default/files/2011_hh_pflegebed_d.pdf> |
| **Long-term care financing** | | | | |
| Total expenditure on long-term care | OECD Statistics : Health : Health expenditure and financing. | Joint OECD, EUROSTAT and WHO Health Accounts SHA Questionnaires (JHAQ) | All financing schemes: Government payments, compulsory schemes, voluntary schemes and household out-of-pocket payments. Current expenditure on health in only long-term care (health) function. All providers: hospitals, residential long-term care facilities, providers of ambulatory care, providers of ancillary services, retailers and other providers of medical goods, providers of preventive care, providers of health care system administration and financing, rest of the economy, rest of the world, providers unknown. | N/A |
| Public expenditure on long-term care (government and compulsory schemes) | OECD Statistics : Health : Health expenditure and financing. | Joint OECD, EUROSTAT and WHO Health Accounts SHA Questionnaires (JHAQ) | Only government payments and compulsory schemes as financing schemes. Current expenditure on health in only long-term care (health) function. All providers: hospitals, residential long-term care facilities, providers of ambulatory care, providers of ancillary services, retailers and other providers of medical goods, providers of preventive care, providers of health care system administration and financing, rest of the economy, rest of the world, providers unknown. | N/A |
| Household out-of-pocket payments on long-term care | OECD Statistics : Health : Health expenditure and financing. | Joint OECD, EUROSTAT and WHO Health Accounts SHA Questionnaires (JHAQ) | Only household out-of-pocket payments as financing schemes. Current expenditure on health in only long-term care (health) function. All providers: hospitals, residential long-term care facilities, providers of ambulatory care, providers of ancillary services, retailers and other providers of medical goods, providers of preventive care, providers of health care system administration and financing, rest of the economy, rest of the world, providers unknown. | N/A |
| Projected public expenditure on long-term care, as % of GDP | **Germany, Italy, Norway (2030-2060):**  European Commission Directorate-General for Economic Financial Affairs. The 2021 ageing report : economic & budgetary projections for the EU Member States (2019-2070). Publications Office; 2021.  **United Kingdom (2030-2060):** European Commission Directorate-General for Economic Financial Affairs. The 2015 ageing report : economic & budgetary projections for the EU Member States (2013-2060). 2015. | **Germany, Italy, Norway (2030-2060):**  European Commission (DG ECFIN), Eurostat (EUROPOP2019), EPC (AWG)  **United Kingdom (2030-2060):** Commission Services (DG ECFIN), Eurostat (EUROPOP2013), EPC (AWG) | **Germany, Italy, Norway, United Kingdom (2030-2060):** The term "LTC services" according to SHA refers to the organisation and delivery of a broad range of services and assistance to people who are limited in their ability to function independently on a daily basis over an extended period of time. The services may be provided in a variety of settings including institutional, residential – i.e. in supported living arrangements, other than nursing homes – or home care. LTC comprises a mix of both health and social components pertaining to both health care and social care sectors  Public expenditure is categorized as expenditure by government and social health insurance as well as compulsory contributory health care financing schemes | **Switzerland (2030-2050):** Data for Switzerland is not available in the The 2021 Ageing Report : economic & budgetary projections for the EU Member States (2019-2070). Long-term care expenditure predictions for Switzerland are original data from: *Eling M, Elvedi M. Die Zukunft der Langzeitpflege in der Schweiz [The future of long-term care in Switzerland]. EconStor: University of St.Gallen, Institute of Insurance Economics; 2019.*  GDP predictions used for the Eling & Elvedi 2019 predictions are from: *Brändle T, Colombier C, Philipona A, Eidgenössisches Finanzdepartement. Langfristperspektiven der öffentlichen Finanzen in der Schweiz [Long-Term Perspectives of Public Finances in Switzerland]. 2016.*  The provision of long-term care in Switzerland is based on three pillars: informal care, inpatient care in retirement and nursing homes, and outpatient care provided by regional Spitex organizations.  Using reference scenario, public expenditure on long-term care was calculated using data from *Eling and Elvedi* 2019's method 1 reference scenario table 16 and table 15:  Predicted public expenditure as % of GDP = [(total projected federal expenditure on long-term care of year + total projected social insurance expenditure on long-term care of year + other projected official financing of year) / total projected long-term care expenditure of year] * total projected long-term care expenditure as % of GDP |
| **Long-term care service delivery** | | | | |
| Long-term care recipients in institutions (other than hospitals) | OECD Statistics : Health : Long-term care resources and utilization. | **Switzerland (2005-2019):** Federal Statistical Office  **Germany (2005-2019):** Federal Ministry of Health, Association of Private Health Insurance, Federal Statistical Office  **Italy (2005-2019):** Ministry of Health - Health Information System  **Norway (2005):** Statistics Norway, Official Statistics of Norway: Nursing and Care Statistics. Annual survey of municipal services (KOSTRA)  **Norway (2010-2015):** IPLOS register. National statistics produced by Statistics Norway  **Norway (2019):** The Municipal Patient and User Registry, Norwegian Directorate of Health | People receiving formal (paid) long-term care in institutions (other than hospitals). Note: The services received by long-term care recipients can be publicly or privately financed. Long-term care institutions refer to nursing and residential care facilities which provide accommodation and long-term care as a package. They include specially designed institutions or hospital-like settings where the predominant service component is long-term care and the services are provided for people with moderate to severe functional restrictions. Inclusion: (1) Persons who receive long-term care by paid long-term care providers, including non-professionals receiving cash payments under a social programme, (2) Recipients of cash benefits such as consumer-choice programmes, care allowances or other social benefits which are granted with the primary goal of supporting individuals with long-term care needs based on an assessment of needs. Exclusion: (1) Persons receiving long-term care in hospitals, (2) Disabled persons of working age who receive income benefits or benefits for labour market integration without long-term care services, (3) Persons who need help only with instrumental activities of daily living (IADL), that is, receiving only long-term social care as defined under the Health Accounts questionnaire.  SHA Classification of Residential Long-term Care:  The category of residential long-term care facilities comprises establishments that are primarily engaged in providing residential long-term care that combines nursing, supervisory or other types of care as required by the residents. In these establishments, a significant part of the production process and the care provided is a mix of health and social services, with the health services being largely at the level of nursing care, in combination with personal care services. The medical components of care are, however, much less intensive than those provided in hospitals.  SHA Classification of Nursing Care Facilities:  Long-term nursing care facilities is a subcategory of residential long-term care that comprises establishments that are primarily engaged in providing inpatient nursing and rehabilitative services for long-term care patients. The care is generally provided for an extended period of time to individuals requiring nursing care. These establishments have a permanent core staff of registered or licensed practical nurses that, along with other staff, provide nursing care in combination with personal care. They provide predominantly long-term care, but also occasionally acute health care and nursing care in conjunction with accommodation and other types of social support, such as assistance with day-to-day living tasks and assistance towards independent living. Included are various establishments that provide long-term care involving regular basic nursing care to chronically ill, frail, disabled or convalescent persons or senile persons placed in an inpatient institution. The exact classification in the corresponding types of institutions (skilled nursing care facilities, residential mental retardation facilities, other residential long-term care facilities) depends on the country-specific division of labour in the care process, especially in long-term care and rehabilitation. | **Norway (2005-2019):** Recipients of short-term care in institutions are included in the figures for recipients of long term care in institutions and not included among the recipients of long term care at home.  **United Kingdom (2019):**  Data for long-term care recipients in institutions was unavailable on OECD Statistics for the UK. UK data to match this indicator was sourced from *carehome.co.uk Care home stats: number of settings, population & workforce.* There is no UK-wide dataset as each country is responsible for its own data collection. This means there are discrepancies in the ways data is collected and reported. The figures should be viewed as estimates. Data on carehome.co.uk was gathered from the Office of National Statistics for England and Wales, Public Health Scotland and Department of Health Ireland.  The proportion of recipients in institutions as a percent of total population was calculated using population data from the World Bank. |
| Beds in residential facilities, n (per 1000 population aged 65 and over) | OECD Statistics : Health : Long-term care resources and utilization. | **Switzerland (2005-2019):** Federal Statistical Office  **Germany (2005-2019):**  Federal Statistical Office  **Italy (2005-2019):**  Ministry of Health, Office of Statistics  **Norway (2005-2019):**  Statistics Norway  **United Kingdom (2005-2019):**  Care Quality Commission Database (England), Department for Health (Northern Ireland), Scottish Care Homes Census (Scotland), Health Statistics Wales (Wales) | Residential long-term care facilities comprise establishments primarily engaged in providing residential long-term care that combines nursing, supervisory or other types of care as required by the residents. In these establishments, a significant part of the production process and the care provided is a mix of health and social services, with the health services being largely at the level of nursing care, in combination with personal care services. The medical components of care are, however, much less intensive than those provided in hospitals. Inclusion (1) Long-term nursing care facilities (SHA Classification: This subcategory comprises establishments that are primarily engaged in providing inpatient nursing and rehabilitative services for long-term care patients. The care is generally provided for an extended period of time to individuals requiring nursing care. These establishments have a permanent core staff of registered or licensed practical nurses that, along with other staff, provide nursing care in combination with personal care. They provide predominantly long-term care, but also occasionally acute health care and nursing care in conjunction with accommodation and other types of social support, such as assistance with day-to-day living tasks and assistance towards independent living. Included are various establishments that provide long-term care involving regular basic nursing care to chronically ill, frail, disabled or convalescent persons or senile persons placed in an inpatient institution. The exact classification in the corresponding types of institutions (skilled nursing care facilities, residential mental retardation facilities, other residential long-term care facilities) depends on the country-specific division of labour in the care process, especially in long-term care and rehabilitation), (2) Other residential long-term care facilities (SHA Classification: This category includes the provision of residential and health care services in organisations classified neither as long-term nursing care facilities , nor as mental health and substance abuse facilities. This includes specialised non-mental residential facilities, as, for example, geriatric rehabilitation facilities that do not fulfil the criteria for geriatric hospitals). Exclusion (1) Beds in hospitals dedicated to long-term care, (2) Beds in residential settings such as adapted housing that can be considered as people’s home. | N/A |
| Long-term care recipients at home, n (% of total population) | OECD Statistics : Health : Long-term care resources and utilization. | **Switzerland (2005):** Federal Office of Social Insurance  **Switzerland (2010-2019):** Federal Statistical Office  **Germany (2005-2019):** Federal Ministry of Health, Association of Private Health Insurance, Federal Statistical Office  **Italy (2005-2019):** Ministry of Health - Health Information System  **Norway (2005):** Statistics Norway, Official Statistics of Norway: Nursing and Care Statistics. Annual survey of municipal services (KOSTRA).  **Norway (2010-2019):** IPLOS register, Statistics Norway  **United Kingdom (2005):** Department of Health in England | People receiving formal (paid) long-term care at home. [Please refer to definition of formal long-term care in the 'total nurses and personal carers' indicator within the long-term care workforce section] Note: The services received by long-term care recipients can be publicly or privately financed. Long-term care at home is provided to people with functional restrictions who mainly reside at their own home. It also applies to the use of institutions on a temporary basis to support continued living at home - such as in the case of community care and day care centres and in the case of respite care. Home care also includes specially designed or adapted living arrangements for persons who require help on a regular basis while guaranteeing a high degree of autonomy and self-control. Inclusion: (1) Persons who receive long-term care by paid long-term care providers, including non-professionals receiving cash payments under a social programme, (2) Recipients of cash benefits such as consumer-choice programmes, care allowances or other social benefits which are granted with the primary goal of supporting individuals with long-term care needs based on an assessment of needs. Exclusion: (1) Disabled persons of working age who receive income benefits or benefits for labour market integration without long-term care services, (2) Persons who need help only with instrumental activities of daily living (IADL), that is, receiving only long-term social care as defined under the Health Accounts questionnaire. | **United Kingdom (2005):** Estimates are for England only  **United Kingdom (2010-2019):** Data for United Kingdom not available on OECD Statistics.  **United Kingdom (2019):**  Data for long-term care recipients at home was unavailable on OECD Statistics for the UK past 2005. UK data to match this indicator was sourced from *homecare.co.uk Home care facts and stats: number of providers, service users & workforce.*  There is no UK-wide dataset as each country is responsible for its own data collection. This means there are discrepancies in the ways data is collected and reported. The figures should be viewed as estimates. Data on homecare.co.uk was gathered from the National Audit Office for England, StatsWales, Public Health Scotland and Department of Health Ireland.  The proportion of recipients at home as a percent of total population was calculated using population data from the World Bank. |
| **Long-term care workforce** | | | | |
| Total nurses and personal carers | OECD Statistics : Health : Long-term care resources and utilization : Long-term care workers. | **Switzerland (2005):** Federal Statistical Office, Federal Office of Social Insurance  **Switzerland (2010-2019):** Federal Statistical Office  **Germany (2005-2019):** Federal Statistical Office  **Norway (2010-2019):** Statistics Norway | Long-term care workers are individuals who provide care to long-term care recipients (all ages). Formal LTC workers include the following occupations and categories: (1) Nurses, as defined by the ISCO-08 classification (2221 for Professional nurses and 3221 for Associate professional nurses), providing long-term care in private homes or in LTC institutions (other than hospitals). Inclusion: (i) Persons who have completed their studies/education in nursing and who are licensed to practice (including both professional nurses and associate/practical/vocational nurses), (ii) Salaried and self-employed nurses delivering services at home or in LTC institutions (other than hospitals); (iii) Foreign nurses licensed to practice and actively practising in the country; (iv) Nurses providing long-term care to patients affected by dementia and/or Alzheimer’s disease. Exclusion: (i) Students who have not yet graduated; (ii) Nursing aids/assistants and care workers who do not have any recognised qualification/certification as a licensed nurse; (iii) Nurses working in administration, research, and in other posts that exclude direct contact with the patients; (iv) Unemployed nurses and retired nurses; (v) Nurses working abroad; (vi) Nurses providing social services; vii) Psychiatric nurses. (2) Personal care workers (caregivers) include formal workers providing LTC services at home or in institutions (other than hospitals) and who are not qualified or certified as nurses. As per the definition in the ISCO-08 classification (5322 for Home-based personal care workers and 5321 for Personal care assistants), personal care workers are defined as people providing routine personal care, such as bathing, dressing, or grooming, to persons who are in need of such care due to effects of ageing, illness, injury, or other physical or mental conditions, in private homes and or in institutions (other than hospitals). Inclusion: (i) Nursing aids/assistants and care workers providing LTC services, who do not have any recognised qualification/certification in nursing, (ii) Family members, neighbours or friends employed (i.e., under a formal contractual obligation and/or declared to social security systems as caregiver) by the care recipient, or person/agency representing the care recipient, and/or by public care services and private care service companies, to provide the care services to the person in need for care. Exclusion: (i) Informal caregivers receiving income support or other cash payments from the care recipient as part of cash programmes and/or consumer-choice programmes, but who are not formally employed, or paid for, by the care recipient (or person/agency representing the care recipient, including providers/organisations, such as public social care services and private care service companies), (ii) Unemployed and retired caregivers, (iii) Caregivers working abroad, (iv) Caregivers in assessment teams employed to evaluate care needs and other persons employed in administrative positions, (vi) Social workers/community workers. | **Italy (2005-2019):** Data for Italy not available on OECD Statistics for long-term care workers at home, and therefore also not for total long-term care workers.  **Norway (2005):** Data unavailable.  **United Kingdom (2005-2019):** Data for United Kingdom not available on OECD Statistics for long-term care nurses, and therefore also not for total long-term care workers.  **United Kingdom (2020):** Estimates are for England only. Data for England was sourced from *Skills for Care (2021). The state of the adult social care sector and workforce in England: 2021*. This is a Skills for Care estimate for the estimated number of adult social care jobs in direct care in England |
| Total nurses and personal carers working in long-term care institutions | OECD Statistics : Health : Long-term care resources and utilization. | **Switzerland (2005-2019):** Federal Statistical Office  **Germany (2005-2019):** Federal Statistical Office  **Italy (2005-2019):** Istat Survey on "Residential Care Institutions"  **Norway (2010-2019):** Statistics Norway | Long-term care institutions herein refer to nursing and residential care facilities which provide accommodation and long-term care as a package. They refer to specially designed institutions or hospital-like settings where the predominant service component is long-term care and the services are provided for people with moderate to severe functional restrictions. Inclusion: (1) Nurses and personal carers providing LTC services in nursing and residential care facilities dedicated to long-term nursing care. Exclusion: (1) Nurses and personal carers providing LTC services in institutions used on a temporary basis to support continued living at home, (2) such as community care, day care centres and respite care, (3) Nurses and personal carers providing LTC services in specially designed or adapted living arrangements for persons who require help on a regular basis while guaranteeing a high degree of autonomy and self-control (defined as home), (3) Nurses and personal carers providing LTC services in hospitals.  [Please refer to definition of total nurses and personal carers in the 'total nurses and personal carers' indicator and 'long-term care recipients in institutions (other than hospitals)' for the SHA classification of residential long-term care facilities and nursing care facilities] | **United Kingdom (2005-2019):** Data for United Kingdom not available on OECD Statistics for long-term care nurses, and therefore also not for total long-term care workers.  **United Kingdom (2020):** Data for England was sourced from *carehome.co.uk Care home stats: number of settings, population & workforce.* |
| Total nurses and personal carers working in long-term home care | OECD Statistics : Health : Long-term care resources and utilization. | **Switzerland (2005-2019):** Federal Statistical Office  **Germany (2005-2019):** Federal Statistical Office | Long-term care at home is provided to people with functional restrictions who mainly reside at their own home. It also applies to the use of institutions on a temporary basis to support continued living at home - such as in the case of community care and day care centres and in the case of respite care. Home care also includes specially designed or adapted living arrangements (for instance, sheltered house) for persons who require help on a regular basis while guaranteeing a high degree of autonomy and self-control, and supportive living arrangements.  [Please refer to definition of total nurses and personal carers in the 'total nurses and personal carers' indicator] | **Italy (2005-2019):** Data not available  **Norway (2005-2019):** Data not available  **United Kingdom (2005-2019):** Data for United Kingdom not available on OECD Statistics for long-term care nurses, and therefore also not for total long-term care workers.  **United Kingdom (2019):**  Data for long-term care workforce at home was unavailable on OECD Statistics for the UK. UK data to match this indicator was sourced from *homecare.co.uk Home care facts and stats: number of providers, service users & workforce.* There is no UK-wide dataset as each country is responsible for its own data collection. This means there are discrepancies in the ways data is collected and reported. The figures should be viewed as estimates. Data on homecare.co.uk was gathered from the Skills for Care for England and Northern Ireland, Social Care Wales and Scottish Social Services Council.  The proportion of nurses and personal carers working in long-term home care as a percent of the population 65 and over was calculated using population data from the World Bank. |
| Proportion of informal carers among population aged 50 and over | OECD Health at a Glance 2011, OECD Health at a Glance 2013, OECD Health at a Glance 2017, OECD Health at a Glance 2021 | **Switzerland (2007-2019):** 2004-2006, 2010, 2015, and 2019-2020 Survey of Health, Ageing and Retirement in Europe (SHARE)  **Germany (2007-2019):** 2004-2006, 2010, 2015, and 2019-2020 Survey of Health, Ageing and Retirement in Europe (SHARE)  **Italy (2007-2019):** 2004-2006, 2010, 2015, and 2019-2020 Survey of Health, Ageing and Retirement in Europe (SHARE)  **United Kingdom (2007):** The 2007 BHPS survey for the United Kingdom  **United Kingdom (2010):** The 2009 BHPS survey for the United Kingdom  **United Kingdom (2015-2019):** The 2015 and 2017 United Kingdom English Longitudinal Study of Ageing (ELSA) | Informal carers are defined as people providing any help to older family members, friends and people in their social network, living inside or outside their household, who require help with everyday tasks. The data presented here relate only to the population aged 50 and over. | **Norway (2007-2019):** Data unavailable  **Norway (2019):** Data for proportion of informal carers among population aged 50 and over is unavailable for Norway. Data for Norway represents a different indicator, percent of unpaid care work among among persons 16 years and over. This data was sourced from *Statistics Norway. Health : Health Services : Unpaid welfare work, survey on living conditions.* |
